# A Provenance-Driven Trust Framework with Physics-Consistent Validation for Secure Wireless Sensor Networks

**DOI:** 10.3390/s26185695

**Published:** 2026-09-08

**Authors:** Eman Abouelkheir

**Affiliations:** Department of Information Technology, College of Computer, Qassim University, Buraidah 51411, Saudi Arabia; e.abouelkheir@qu.edu.sa

**Keywords:** wireless sensor networks, sensor data aggregation, provenance, trust management, intrusion detection, physics consistency, blockchain anchoring, cyber-physical security, wireless communication

## Abstract

Wireless sensor networks (WSNs) play a critical role in cyber-physical applications such as industrial monitoring, environmental sensing, and critical infrastructure management. In these environments, security mechanisms must not only detect malicious activities but also explain how compromised measurements propagate through sensing, aggregation, and decision processes while operating under stringent resource constraints. Existing approaches typically address intrusion detection, trust management, provenance analysis, or blockchain-based integrity independently, providing limited support for integrated and explainable security. This paper presents PhyProvTrust-WSN, a physics-aware framework that combines physics-consistency validation, dynamic provenance graphs, evidence-based trust propagation, multi-factor risk fusion, and selective evidence anchoring to improve the transparency and auditability of secure sensor data aggregation. The framework models sensing, forwarding, aggregation, validation, and response events as a bounded provenance directed acyclic graph (DAG), enabling causal tracing of suspicious activities while maintaining low memory and communication overhead. A weighted risk fusion mechanism integrates anomaly evidence, domain-consistency assessment, trust evolution, and inherited provenance risk to support explainable security decisions. Rather than continuously recording all events, only high-risk or decision-relevant evidence hashes are anchored to a permissioned audit layer, reducing storage and communication costs. To avoid overclaiming, the proposed framework is evaluated using a hybrid methodology that combines attack-labeled WSN datasets, real sensor measurements for physics-consistency validation, and simulation-based overhead analysis. The results demonstrate that the integrated framework provides strong detection capability while improving explainability, supporting root-cause analysis, and maintaining bounded communication and storage overhead suitable for resource-constrained WSN deployments.

## 1. Introduction

Wireless sensor networks (WSNs) provide low-cost and distributed sensing for environments where continuous observation is required but wired infrastructure is impractical. They support environmental monitoring, industrial automation, structural health monitoring, smart energy systems, precision agriculture, intelligent transportation, and emergency-response applications [1,2,3,4]. Their practical advantage is also the source of their security exposure: sensor nodes are resource constrained, communicate over open wireless channels, and are often deployed in unattended or harsh physical environments.

In mission-critical WSNs, the security problem is not limited to preventing packet loss or detecting a single malicious packet. A stealthy adversary can inject physically plausible but gradually biased measurements, compromise a cluster head, selectively forward packets, replay old readings, or manipulate aggregated summaries. Such attacks may remain below conventional statistical thresholds while still affecting the physical process being monitored. Consequently, the relevant question becomes: how did a malicious measurement move through the sensing, routing, aggregation, and decision chain, and what evidence supports the response?

Traditional security mechanisms address important but incomplete aspects of this question. Secure routing and aggregation protocols improve confidentiality and integrity of data flows [5,6,7,8,9,10]. Machine-learning intrusion detection systems (IDSs) classify normal and abnormal traffic using labeled traces [11,12,13,14,15,16]. Trust-management schemes estimate node reliability and support routing or access decisions [17,18,19,20,21,22]. Blockchain-based approaches improve tamper resistance and distributed accountability in IoT and WSN settings [23,24,25,26,27,28,29,30]. Yet these lines of work rarely combine causal evidence, physical plausibility, trust propagation, and low-overhead auditability in a single WSN-aware model.

The central observation of this study is that trust scores alone hide propagation effects. A single scalar score may identify a suspicious node, but it does not show which measurement, route, aggregation step, or decision was affected. Likewise, blockchain can preserve an immutable record, but it cannot make an incorrect sensor reading physically correct. Provenance graphs are therefore used here as an evidence layer that links source, transformation, decision, action, and outcome [31,32,33,34,35,36]. When combined with physics-consistent validation and dynamic trust propagation, provenance can support accountability and explainable response in resource-constrained WSNs.

This paper proposes PhyProvTrust-WSN, a physics-consistent provenance and trust propagation framework for secure sensor data aggregation. The framework is intentionally conservative: it does not claim universal protection for all WSN threats or full real-world deployment. Instead, it provides a reproducible architecture and evaluation protocol that measures detection, false alarms, root-cause traceability, trust stability, communication overhead, energy cost, and scalability. The design follows the principle that blockchain should be used selectively for evidence anchoring, not as a continuous storage layer for all sensor traffic.

The contributions are:

1. A physics-consistent provenance graph model tailored to clustered WSN data aggregation.
2. A multi-factor risk score combining anomaly evidence, domain-violation risk, trust risk, and inherited provenance risk.
3. A trust propagation mechanism that updates node reliability through weighted evidence dependencies rather than isolated behavioral counters.
4. A selective evidence-anchoring rule that records compact hashes for high-risk events while avoiding Full Blockchain logging.
5. A cautious hybrid evaluation design using WSN attack traces, real sensor readings, and simulation-based overhead analysis.

## 2. Related Work

### 2.1. Secure Routing and Aggregation in WSNs

WSN routing and clustering protocols must balance reliability, security, and energy consumption. Foundational routing and clustering surveys discuss the limited processing power, dynamic topology, and energy constraints that define WSN design [1,2,3,4]. Secure routing research has shown that WSNs are vulnerable to sinkhole, wormhole, Sybil, selective forwarding, and blackhole attacks, especially when nodes are unattended and routing decisions depend on local claims [5,6]. Secure aggregation mechanisms reduce exposure during in-network processing, but most of them emphasize confidentiality or integrity of aggregation values rather than causal explanation after a wrong aggregate is produced [7,8,9,10].

### 2.2. Machine-Learning Intrusion Detection

Machine-learning IDSs have become a common response to WSN attacks because classifiers can learn patterns from labeled traffic. WSN-DS remains a widely used dataset for DoS-oriented WSN intrusion detection, while WSN-BFSF provides a more recent attack-labeled benchmark containing blackhole, flooding, selective forwarding, and normal traffic [11,12]. Recent studies and surveys report strong performance for ensemble learning, deep learning, and feature-selection methods on these datasets [13,14,15,16]. However, high classification accuracy on a labeled benchmark does not automatically provide root-cause evidence, physical plausibility validation, or energy-aware response.

### 2.3. Trust Management

Trust-management systems estimate the reliability of nodes using direct observations, recommendations, packet-forwarding behavior, residual energy, and context-aware attributes [17,18,19,20,21,22]. Hierarchical and clustered trust protocols are particularly relevant to WSNs because many deployments use cluster heads to reduce communication cost [19,20]. Recent work has also explored machine-learning-based adaptive trust generation [21] and comparative evaluation of trust frameworks [22]. The limitation is that trust is often represented as a scalar node-level value. Such a value is useful for routing or isolation decisions, but it does not explain how an event affected downstream aggregations.

### 2.4. Blockchain and Lightweight Auditability

Blockchain has been proposed for IoT and WSN security because it offers distributed integrity, non-repudiation, and auditability [23,24,25,26,27,28,29,30]. In resource-constrained sensor networks, however, continuous ledger updates can increase communication, storage, and latency overhead. Recent surveys and blockchain-enabled WSN works repeatedly emphasize the need for lightweight and selective designs [24,25,26,27,28,29,30]. PhyProvTrust-WSN therefore treats blockchain as an evidence-anchoring option: it stores compact hashes for high-risk or decision-relevant events rather than raw sensor data or complete histories.

### 2.5. Provenance and Causal Security

Data provenance records the origin, movement, and transformation history of data objects. Secure provenance systems were developed to prevent history forgery and support accountability [31]. In WSNs, provenance is particularly important because a base station receives values that may have been forwarded, compressed, aggregated, or transformed by several intermediate nodes [32,33,34]. Recent IoT provenance surveys highlight integrity, confidentiality, accountability, and resource efficiency as core requirements [35,36]. Provenance-based intrusion detection also shows that graph-structured history can expose attacks that are difficult to identify from isolated log entries [37,38,39].

Unlike prior blockchain-enabled WSN frameworks that log continuous traffic, PhyProvTrust-WSN treats blockchain as an evidence-anchoring mechanism rather than a runtime security engine. Likewise, unlike classifier-only IDS approaches, it does not treat attack detection as the final objective; instead, it uses anomaly evidence as one component in a broader causal, physics-consistent, and trust-aware decision process. This positioning is central to the contribution because it reduces overclaiming and clarifies that the proposed model is an auditable security reasoning layer for sensor data aggregation. Table 1 presents PhyProvTrust-WSN against the major related research directions.

Quantitatively, the novelty is expressed as an added-evidence operating point rather than as a universal classification gain. Using the directly comparable results reported later in the manuscript, selective anchoring reduces relative communication overhead from 1.60 for Full Blockchain to 1.18 for PhyProvTrust-WSN, i.e., (1.60–1.18)/1.60 = 26.3%. Against the Random Forest IDS, the proposed framework preserves the same 99.4% accuracy on WSN-DS while increasing recall from 97.1% to 97.4%, and on WSN-BFSF it preserves 99.3% accuracy and 97.9% F1 while reducing FPR from 0.5% to 0.2%. These comparisons make the contribution concrete: the framework primarily adds traceability, physics-aware reasoning, trust evolution, and selective auditability while retaining classifier-level detection performance and lowering audit overhead relative to continuous logging.

## 3. Research Gap and Motivation

The reviewed literature reveals a gap that is both methodological and practical. Existing WSN security mechanisms usually optimize one target: packet delivery, classification accuracy, node reputation, or ledger integrity. Mission-critical WSNs require a combined target: the system must detect suspicious behavior, evaluate whether measurements are physically plausible, explain how the suspicious event propagated, and record only the evidence necessary for later audit.

The gap can be stated as follows: current WSN security frameworks do not jointly integrate physics-consistent validation, provenance-based causal modeling, evidence-based trust propagation, and selective tamper-resistant anchoring under explicit energy and communication constraints. This gap is important because mission degradation may occur even when individual packets appear syntactically valid and when statistical anomalies are weak.

### 3.1. Motivating Scenario

Consider a temperature-monitoring WSN in an industrial process. A compromised sensor gradually increases its reported temperature by a small amount that remains below a fixed anomaly threshold. A cluster head aggregates the biased value with neighboring readings, and the base station triggers a control response. A standard IDS may miss the drift, a trust score may decrease only slowly, and a blockchain system may immutably store the manipulated reading. A provenance-aware approach can instead trace which event influenced the aggregate, compare the reading with neighbor and temporal constraints, update trust through the affected path, and anchor the relevant evidence if the risk exceeds a decision threshold.

### 3.2. Design Requirements

- Explainability: every alert should be linked to a provenance path, not only a classifier label.
- Physics consistency: measurements should be checked against temporal, spatial, and domain constraints.
- Energy awareness: provenance metadata and audit records must be compact and selectively retained.
- Trust stability: trust updates should avoid extreme oscillation under transient noise.
- Evaluation realism: experiments should report false alarms, energy, network lifetime, communication overhead, and ablation results, not accuracy alone.

## 4. System Model

**Figure 1:**
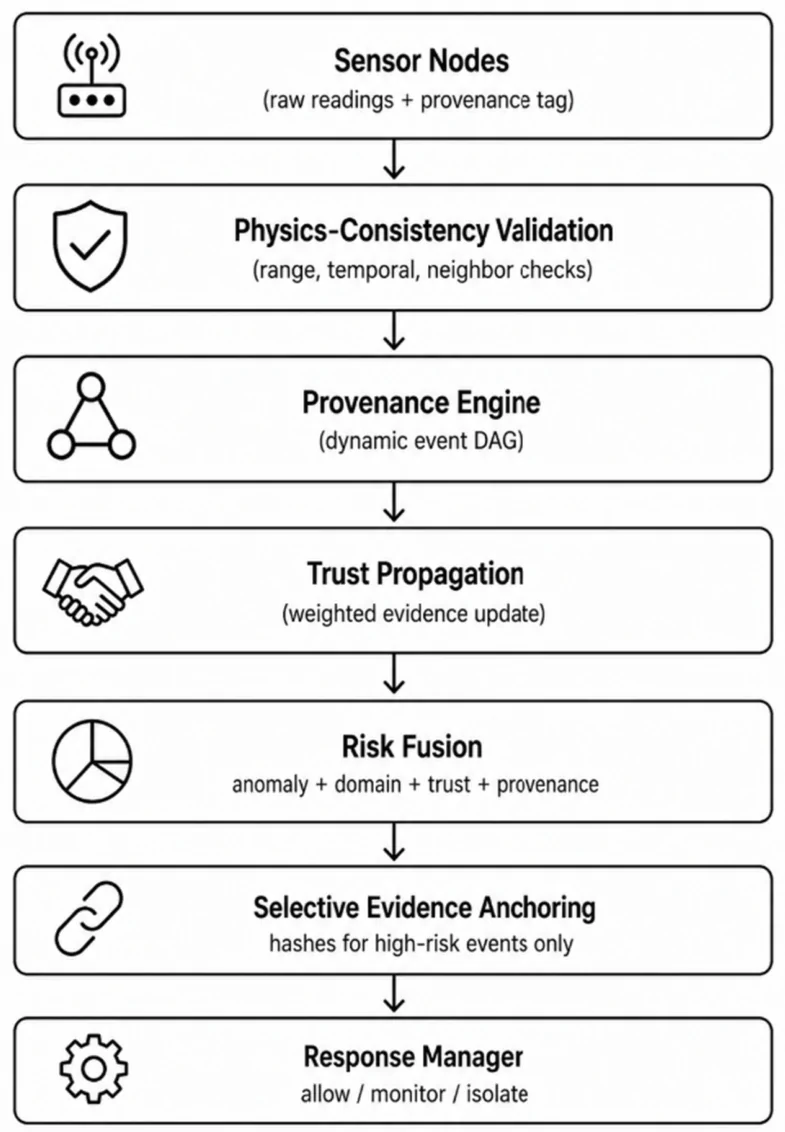
Layered architecture of PhyProvTrust-WSN.

We consider a clustered WSN deployed in a cyber-physical monitoring environment. The network contains sensor nodes, cluster heads, a base station, edge validators, and a lightweight permissioned evidence layer. The system architecture is shown in Figure 1.

### 4.1. Entities

Table 2 show the proposed System entities and responsibilities.

### 4.2. Communication and Retention Assumptions

Sensor readings are transmitted through multi-hop or cluster-based routes. Provenance metadata are piggybacked in compressed form: event identifier, parent event references, timestamp, compact dependency weights, and payload hash. Nodes retain only a sliding window of recent provenance edges. Full raw histories are not stored at sensor nodes. This bounded retention is essential for memory-constrained deployments.

### 4.3. Provenance Graph

**Figure 2:**
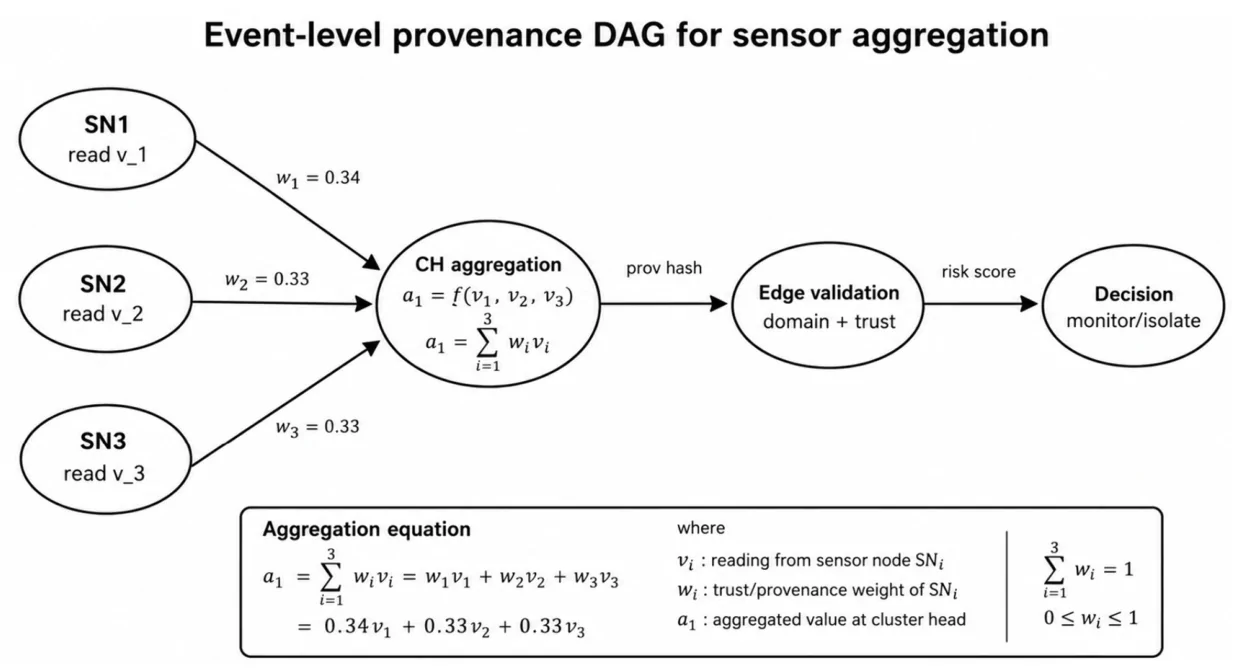
Example event-level provenance DAG for sensor aggregation.

To provide explainable and auditable security reasoning, the proposed framework represents the event history at time t as a dynamic provenance graph, $G_{t}=\left(V_{t},E_{t},W_{t},X_{t}\right)$ where $V_{t}$ denotes the set of sensing, forwarding, aggregation, physics-validation, trust-update, and response events, while $E_{t}$ represents the directed causal dependencies among these events. The matrix $W_{t}$ stores normalized dependency weights that quantify the influence of parent events on subsequent events and are used for evidence-based trust propagation. The attribute set $X_{t}$ contains event metadata, including the node identifier, timestamp, sensor measurement, event type, local trust value, and payload hash. Unlike conventional provenance approaches that primarily record event lineage [31,32,33,34,35,36], the proposed model incorporates weighted dependencies to support trust propagation, multi-factor risk fusion, and attack attribution while maintaining a bounded sliding-window directed acyclic graph (DAG) suitable for resource-constrained WSNs [32,33,34,35,36,37,38,39]. Figure 2 illustrates the event-level provenance graph employed in the proposed framework.

## 5. Threat Model

The adversary is computationally bounded and can compromise a minority of sensor nodes or cluster heads. The attacker may modify readings, replay packets, selectively forward messages, create Sybil identities, manipulate aggregated summaries, or poison reputation signals. The attacker aims to degrade the mission while avoiding immediate detection. The model excludes physical destruction of nodes, high-power jamming, full network partition, compromise of a majority of audit validators, and cryptographic primitive breakage. Table 3 shows the Threat coverage, supporting literature, and evidence requirements of the proposed system.

## 6. Proposed Framework

PhyProvTrust-WSN processes each event through five stages: measurement preprocessing, physics-consistent validation, provenance graph update, trust propagation, and risk-fusion-based response. Evidence anchoring is triggered only when the final score exceeds a logging threshold or when a response decision must be audited.

Operationally, each event enters the framework as a timestamped measurement with node identity, payload metadata, and available parent-event references. The anomaly stage outputs A_e, the physics-consistency stage outputs D_e, the provenance stage updates G_t and the dependency weights w_{uv}, and the trust stage updates T_i(t). These quantities are then converted to trust and provenance risks and combined by the risk-fusion stage to produce R_e, which is the input to the Allow/Monitor/Isolate response policy and to the selective anchoring rule. Thus, the output of each stage is explicitly consumed by the next stage without introducing an additional model.

### 6.1. Physics-Consistent Validation

Deployment portability is handled by separating the generic validation interface from the application-specific physical model. For each sensor modality m, the validator supplies an expected-value function f_m(·) and an admissible deviation bound Δ_max,m. Thus, the provenance, trust, risk-fusion, and anchoring stages remain unchanged when the sensing domain changes; only f_m(·) and Δ_max,m require modality-specific calibration. For slowly varying measurements such as temperature, temporal continuity and neighborhood consistency are suitable references, whereas vibration, pressure, mobility, or industrial-process deployments may require different state, rate-of-change, conservation, or process constraints. The framework therefore does not assume that one physical model is universally transferable across cyber-physical domains.

To evaluate the physical plausibility of each sensor measurement before trust updating and provenance propagation, the proposed framework performs a physics-consistent validation process. For a sensor reading $x_{i}\left(t\right)$, the expected measurement ${\hat{x}}_{i}\left(t\right)$ is estimated using a configurable combination of temporal moving-average prediction, neighboring sensor consistency, and application-specific physical range constraints. Similar principles have been widely employed to validate sensor observations in cyber-physical monitoring systems and wireless sensor networks [9,10,40,41].

For reproducibility, the prediction window W_p is defined as the number of preceding valid samples used by the temporal predictor, while the neighborhood set N_i(t) contains spatially or logically adjacent sensors available at time t. Neighbor consistency is computed from the robust neighborhood aggregate (median when at least three neighbors are available; otherwise the mean of the available neighbors). The physical-deviation bound Δ_max is modality specific and is calibrated from benign validation traces rather than fixed globally. For the Intel Berkeley Lab and SensorScope modalities, the bound is obtained from the upper tail of the absolute benign prediction error after the temporal and neighborhood estimates are combined; the selected value is then frozen before evaluation.

The normalized domain-violation score is computed as $D_{i}\left(t\right)=\min\left(1,\frac{|x_{i}\left(t\right)-{\hat{x}}_{i}\left(t\right)|}{\tau_{d}}\right)$, where $\tau_{d}$ denotes the maximum acceptable physical deviation under normal operating conditions. A larger value of $D_{i}\left(t\right)$ indicates a greater inconsistency between the observed and expected measurements and consequently increases the security risk associated with the corresponding event.

Rather than immediately rejecting measurements that exceed the expected physical behavior, the proposed framework treats the domain-violation score as one component of the overall security evidence. Events with elevated $D_{i}\left(t\right)$ are subsequently analyzed through provenance-aware trust propagation and multi-factor risk fusion before a final security decision is reached. This strategy improves robustness against transient environmental fluctuations and measurement noise while reducing unnecessary false alarms caused by legitimate variations in sensor observations [13,21,35].

### 6.2. Evidence-Based Trust Propagation

To capture the cumulative impact of security events over time, each node u maintains a dynamic trust value $T_{u}\left(t\right)\in \left[0,1\right]$, where higher values indicate greater confidence in the node’s behavior. Unlike conventional trust models that rely primarily on direct observations [17,18,19,20,21,22], the proposed framework updates trust using both local evidence and weighted provenance dependencies. The trust update is defined as $T_{u}\left(t+1\right)={clip}_{\left[0,1\right]}\left(\left(1-\lambda \right)T_{u}\left(t\right)+\lambda \sum_{p\in Pa\left(u\right)}w_{pu}T_{p}\left(t\right)-\eta R_{u}\left(t\right)\right)$ where $Pa\left(u\right)$ denotes the set of parent events or nodes in the provenance graph, $w_{pu}$ represents the normalized dependency weight, $R_{u}\left(t\right)$ is the current event risk, λ controls the propagation strength, and η determines the penalty applied to suspicious behavior. The clipping operator, ${clip}_{\left[0,1\right]}\left(z\right)=max\left(0,min\left(1,z\right)\right),$ ensures that trust values remain bounded within the interval $\left[0,1\right]$. The corresponding trust risk is then defined as $TR_{u}\left(t\right)=1-T_{u}\left(t\right),$ where larger values indicate lower confidence in the reliability of the corresponding node. This evidence-driven trust propagation enables suspicious behavior to influence subsequent security decisions through the provenance graph while maintaining stable trust evolution under transient network disturbances [17,18,19,20,21,22,31,32,33,34,35,36,37,38,39].

### 6.3. Provenance Risk

Beyond local trust assessment, the proposed framework quantifies the inherited influence of previously suspicious events through a provenance risk score. For each event v, the provenance risk is computed as $PR_{v}\left(t\right)=\min\left(1,\sum_{p\in Anc\left(v\right)}w_{pv}TR_{p}\left(t\right)\;decay\left(d\left(p,v\right)\right)\right)$, where $Anc\left(v\right)$ denotes the set of ancestor events in the provenance graph, $w_{pv}$ is the normalized dependency weight, and $d\left(p,v\right)$ represents the graph distance between ancestor p and event v. The decay function is defined as $decay\left(d\right)=e^{-\lambda_{d}d},\lambda_{d}>0$, which progressively reduces the influence of distant ancestor events. This exponential attenuation prevents excessive risk accumulation in long provenance chains while preserving the most relevant causal dependencies within the sliding-window graph. Consequently, the provenance risk captures how compromised upstream events propagate their influence to downstream aggregation and decision processes, thereby improving attack attribution and explainability [31,32,33,34,35,36,37,38,39].

### 6.4. Multi-Factor Risk Fusion

The fusion parameters are deployment parameters rather than universal constants. Let w=[α,β,γ,δ]^T denote the non-negative risk weights, with α + β + γ + δ = 1. The coefficients and the monitoring, isolation, and anchoring thresholds are selected on validation data for the target deployment and are frozen before test evaluation. Candidate settings are compared using the deployment objective in Section 7 so that missed attacks, false alarms, energy consumption, and communication overhead are balanced according to application priorities. Consequently, a safety-critical deployment may assign greater cost to false negatives, whereas an energy-limited environmental deployment may favor a more conservative monitoring and anchoring rate. This calibration requirement is explicitly acknowledged as deployment dependent.

**Figure 3:**
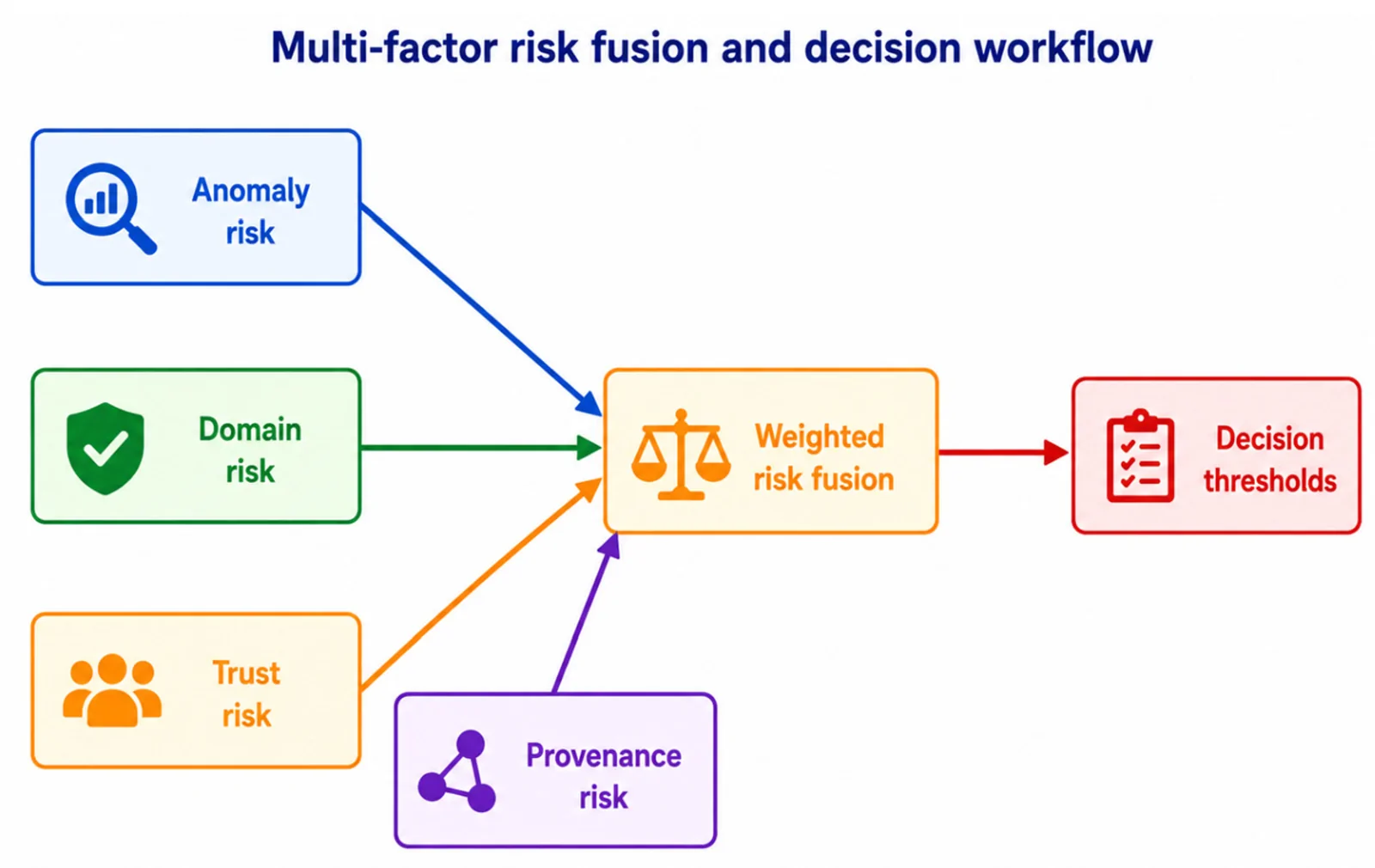
Multi-factor risk fusion and decision workflow.

The final security decision is obtained by integrating complementary sources of evidence into a unified risk score. Specifically, the proposed framework combines anomaly evidence $A_{v}\left(t\right)$, domain-violation risk $D_{v}\left(t\right)$, trust risk $TR_{v}\left(t\right)$, and provenance risk $PR_{v}\left(t\right)$ using the weighted fusion model $S_{v}\left(t\right)=\alpha A_{v}\left(t\right)+\beta D_{v}\left(t\right)+\gamma TR_{v}\left(t\right)+\delta PR_{v}\left(t\right),$ subject to α+β+γ+δ=1,α,β,γ,δ≥0. This multi-factor formulation allows the framework to balance statistical anomaly detection, physical consistency, trust evolution, and causal evidence within a single decision metric. Rather than relying on any individual indicator, the fused score provides a more robust assessment of event reliability and serves as the basis for monitoring, isolation, and selective evidence anchoring described in the subsequent sections. The overall workflow of the risk-fusion process is illustrated in Figure 3. This evidence-driven strategy enhances robustness against stealthy attacks while improving explainability and reducing false alarms in resource-constrained wireless sensor networks [13,14,15,16,17,18,19,20,21,22,31,32,33,34,35,36,37,38,39].

The trust coefficients η and λ, the provenance-decay coefficient γ, and the fusion weights $\alpha_{A},\;\alpha_{D},\;\alpha_{T},\;\alpha_{P}$ are selected on validation data. Candidate settings are evaluated against detection error and operational cost, with the fusion weights constrained to be non-negative and to sum to one. The monitoring, isolation, and anchoring thresholds $\theta_{M},\;\theta_{I},\;\theta_{A}$ are then selected from the validation risk-score distribution and frozen for the test set. This calibration separates parameter selection from the reported test operating point and prevents test-set tuning.

Reproducibility note on the numerical operating point: the archived experiment tables available for this revision contain the final performance outputs but do not contain the run-configuration record from which the exact numerical values of $\Delta_{max},\eta ,\lambda ,\gamma ,\alpha_{A},\alpha_{D},\alpha_{T},\alpha_{P},\theta_{M},\theta_{I},\;and\;\theta_{A}$ can be recovered. Accordingly, these values are not reconstructed from the test results or assigned retrospectively in this revision, because doing so would introduce undocumented test-set tuning. The experimental procedure is therefore stated explicitly: candidate values are selected exclusively on the validation partition, the selected configuration is frozen, and the unchanged configuration is then applied to the test partition. For a fully reproducible release, the exact run-configuration file containing these numerical values must accompany the manuscript/original configuration files.

### 6.5. Selective Evidence Anchoring

To ensure the integrity and auditability of security-critical events while minimizing communication and storage overhead, the proposed framework employs a selective evidence-anchoring strategy. Unlike conventional blockchain-based approaches that continuously record all network events [23,24,25,26,27,28,29,30], the proposed method anchors only events that are considered security relevant. Specifically, if the fused risk score satisfies $S_{v}\left(t\right)\geq \theta_{log},$ or if the event triggers a security response, a compact evidence digest is generated as $H_{v}=Hash(\mathrm{event}\text{\_}\mathrm{id}\;∥\;\mathrm{timestamp}\;∥\;\mathrm{parent}\text{\_}\mathrm{hashes}\;∥\;\mathrm{score}\;∥\;\mathrm{decision}) ,$ where ∥ denotes concatenation. The resulting hash $H_{v}$ is anchored to the permissioned audit layer to provide tamper-evident verification without storing raw sensor measurements or complete provenance histories. By recording only decision-relevant evidence, the proposed approach preserves forensic accountability while significantly reducing blockchain communication, storage, and computational overhead in resource-constrained wireless sensor networks [23,24,25,26,27,28,29,30,31,32].

### 6.6. Response Policy

The final security response is determined according to the fused risk score $S_{v}\left(t\right)$. Three operational thresholds are defined to support progressive mitigation actions. Events satisfying $S_{v}\left(t\right)<\theta_{monitor}$ are regarded as benign and are processed normally. Events with $\theta_{monitor}\leq S_{v}\left(t\right)<\theta_{block}$ are classified as suspicious and are subjected to continuous monitoring together with additional provenance and trust analysis. Finally, events satisfying $S_{v}\left(t\right)\geq \theta_{block}$ are considered high risk, leading to node isolation, traffic quarantine, or exclusion from the aggregation process, depending on the deployment policy.

To improve resilience against transient measurement errors and environmental fluctuations, the proposed framework adopts a reversible trust-management mechanism. Nodes that subsequently exhibit consistent benign behavior gradually recover their trust values through the evidence-based trust propagation process described in Section 6.2, thereby preventing permanent penalties caused by temporary disturbances or false alarms. This adaptive response strategy enhances network reliability while maintaining robust protection against persistent attacks [17,18,19,20,21,22,31,32,33,34,35,36,37,38,39].

## 7. Mathematical Formulation

This section provides a unified mathematical representation of the proposed PhyProvTrust-WSN framework. Each sensing, forwarding, aggregation, validation, trust-update, and response operation is represented as an event within a time-dependent provenance graph. This formulation captures both the attributes of individual events and the causal dependencies through which suspicious information may influence subsequent aggregation and decision-making processes.

For each event, the framework evaluates four complementary and normalized sources of security evidence: anomaly risk, physics-consistency risk, trust risk, and provenance risk. The physics-consistency component measures the deviation between the observed and expected sensor values, whereas the trust component reflects the reliability of the originating or forwarding node. The provenance component captures the inherited influence of suspicious ancestor events, while the anomaly component represents the output of the statistical or machine-learning detection model. These components are combined using non-negative normalized weights to produce a bounded fused risk score.

The resulting risk score provides a common basis for the Allow, Monitor, Isolate, and selective evidence-anchoring decisions. All model parameters, including the physical-deviation bounds, trust coefficients, provenance-decay factor, fusion weights, and operational thresholds, are calibrated exclusively on the validation partition and subsequently frozen before test evaluation. This separation prevents test-set tuning and ensures that the reported operating point reflects the intended deployment procedure.

The formulation is developed under four principal constraints: individual risk components remain within the interval $\left[0,1\right]$; node trust values are bounded through the clipping operator; dependency weights are normalized over the retained parent events; and the provenance history is restricted to a finite sliding window. These constraints ensure numerical stability, bounded memory consumption, and interpretable threshold-based decisions in resource-constrained wireless sensor networks. Table 4 summarizes the notation used throughout the formulation, whereas Table 5 specifies the role and calibration procedure of the principal framework parameters.

### 7.1. Word Equation Editor Forms

**Proposition 1** 
(Bounded Fused Risk)**.** *Let* $A_{v}\left(t\right)$*,* $D_{v}\left(t\right)$*,* $TR_{v}\left(t\right)$*, and* $PR_{v}\left(t\right)$ *denote the anomaly, domain-violation, trust, and provenance risk components, respectively, where* $A_{v}\left(t\right),\;D_{v}\left(t\right),\;TR_{v}\left(t\right),\;PR_{v}\left(t\right)\in \left[0,1\right].$ *Assume that the weighting coefficients satisfy* α,β,γ,δ≥0, α+β+γ+δ=1. *Then, the fused risk score* $S_{v}\left(t\right)=\alpha A_{v}\left(t\right)+\beta D_{v}\left(t\right)+\gamma TR_{v}\left(t\right)+\delta PR_{v}\left(t\right)$ *is bounded within the interval* $0\leq S_{v}\left(t\right)\leq 1.$

**Proof.**  Since $S_{v}\left(t\right)$ is a convex combination of four bounded quantities, its minimum value is attained when all components are equal to zero, while its maximum value occurs when all components are equal to one. Consequently, $0\leq S_{v}\left(t\right)\leq 1.$ Therefore, the fused risk score is always normalized and can be directly employed for threshold-based security decisions without requiring additional normalization. □

**Proposition 2** 
(Bounded Trust Evolution)**.** *Assume that the initial trust value satisfies*  $T_{u}\left(0\right)\in \left[0,1\right],$ *and that trust is updated according to the proposed rule* $T_{u}\left(t+1\right)={clip}_{\left[0,1\right]}\left(z\right),$ *where* ${clip}_{\left[0,1\right]}\left(z\right)=max\left(0,min\left(1,z\right)\right).$ *Then,* $T_{u}\left(t\right)\in \left[0,1\right],\forall t\geq 0.$

**Proof.**  Regardless of the intermediate value z produced by the trust update equation, the clipping operator projects the result onto the closed interval $\left[0,1\right]$. Since the initial trust value belongs to the same interval, repeated application of the update rule preserves this bound for all subsequent iterations. Hence, the trust evolution remains numerically stable throughout network operation. □

#### 7.1.1. Optimization Objective

The proposed framework aims to achieve an effective balance between security performance and resource efficiency by minimizing the following deployment cost function: $\min L=c_{FN}\;FN+c_{FP}\;FP+c_{E}\;E+c_{C}\;C$, where FN and FP denote the numbers of false negatives and false positives, respectively, E represents the energy consumption, and C denotes the communication overhead incurred during monitoring, provenance maintenance, and selective evidence anchoring. The weighting coefficients $c_{FN},c_{FP},c_{E},$ and $c_{C}$ are application-dependent cost parameters that allow the framework to prioritize security accuracy or resource efficiency according to deployment requirements. Consequently, the proposed optimization model can be readily adapted to diverse wireless sensor network applications, including industrial monitoring, environmental sensing, precision agriculture, and other resource-constrained cyber-physical systems.

#### 7.1.2. Sensitivity and Security-Overhead Trade-Off

Sensitivity is interpreted around the frozen validation operating point rather than by retuning on the test data. Increasing θ_A decreases the anchoring ratio ρ and therefore lowers blockchain communication overhead, but it also reduces the number of suspicious events preserved in the audit layer. Lowering θ_M or θ_I increases security sensitivity at the cost of more monitoring or isolation actions and potentially more false alarms.

A local sensitivity analysis is performed by varying one calibrated parameter at a time around its frozen validation value while keeping all remaining parameters and the data partition unchanged. For each risk-fusion weight $\alpha_{k}$, the perturbed weight vector is renormalized to satisfy $\sum_{k}\alpha_{k}=1.$ For the physical-deviation bound $\Delta_{max}$ and the decision thresholds, perturbations are applied multiplicatively relative to the calibrated operating point. Robustness is assessed by examining the resulting variations in the F1-score, false positive rate (FPR), and anchoring ratio ρ. Because the retained experimental archive does not contain the outputs generated for each parameter perturbation, this revision does not report reconstructed numerical sensitivity intervals. Instead, the sensitivity-analysis procedure is specified explicitly so that the analysis can be reproduced once the original run configuration is available.

## 8. Algorithms

### 8.1. Event Processing

The deployment objective is used exclusively for parameter calibration and operating-point selection; it is not optimized during the execution of Algorithm 1 for each incoming event. Specifically, the cost weights $\omega_{FN}$, $\omega_{FP}$, $\omega_{E}$, and $\omega_{C}$ represent the relative priorities assigned to missed attacks, false alarms, energy consumption, and communication overhead, respectively. Validation experiments are used to compare candidate fusion weights and decision thresholds under this objective function, after which a single operating point is selected and frozen. Algorithm 1 subsequently applies these fixed parameters as part of a rule-based online decision workflow. Therefore, Section 7 describes how the deployment configuration is selected, whereas Section 8 describes how the selected configuration is executed on an event-by-event basis.

The proposed framework processes each sensor event through a sequence of validation, provenance construction, trust updating, risk evaluation, and response operations. Algorithm 1 summarizes the event-level processing workflow.
**Algorithm 1.** PhyProvTrust Event Processing**Input:** event e; timestamp and payload metadata; anomaly model; physical-validation configuration; current provenance graph $G_{t}$; trust state T(t); calibrated risk weights and decision thresholds.$\;(node_{id},timestamp,value,\;parent_{ref},\;payload\;hash)$
**Output:** Fused risk score $S_{e}$ and corresponding security decision.   Verify the timestamp consistency and payload integrity of event e.   Compute the anomaly risk $A_{e}$ using the trained anomaly detector or rule-based validation model.   Estimate the expected sensor value ${\hat{x}}_{e}$ and calculate the domain-violation score $D_{e}$.   Insert the event into the sliding-window provenance graph and establish dependency links with its parent events.   Update the local trust value and propagate inherited trust using the weighted provenance dependencies.   Compute the provenance risk $PR_{e}$ from suspicious ancestor events   Calculate the fused risk score $S_{e}$ by combining $A_{e}$, $D_{e}$, $TR_{e}$, and $PR_{e}$.   Apply the predefined decision thresholds to classify the event as **Allow**, **Monitor**, or **Isolate**.   If $S_{e}\geq \theta_{log}$ or the selected response requires auditing, generate and anchor the corresponding evidence hash.

### 8.2. Selective Evidence Anchoring

To reduce blockchain communication and storage overhead, only security-relevant events are recorded in the permissioned audit layer. Algorithm 2 summarizes the selective evidence-anchoring procedure.
**Algorithm 2.** Selective Evidence Anchoring**Input:** Event e, fused risk score $S_{e}$, and response decision $d_{e}$.**Output:** Anchored evidence hash $H_{e}$ and local audit record.   If $S_{e}<\theta_{log}$ and $d_{e}=\mathrm{Allow}$, terminate the procedure without blockchain anchoring.   Construct the compact evidence tuple containing the event identifier, timestamp, parent hashes, discretized risk score, and response decision.   Compute the evidence digest: $H_{e}\left(event_{id},timestamp,parent_{hashs},\;score,\;decision\right)$
   Submit the generated hash to the permissioned audit validators for integrity verification.   Store the returned confirmation identifier within the local audit index for subsequent provenance verification.


$$H_{e}=Hash(\mathrm{event}\text{\_}\mathrm{id}\;∥\;\mathrm{timestamp}\;∥\;\mathrm{parent}\text{\_}\mathrm{hashes}\;∥\;\mathrm{score}\;∥\;\mathrm{decision})$$

### 8.3. Computational Complexity

The practical cost of the provenance DAG is governed by the sliding-window size and retained dependency density. Let W be the maximum number of events retained in the active window, $\bar{d}$ the average number of retained parent edges per event, and E_W the number of active dependency edges. Then $E\_W\;\leq \;W\;\bar{d}$, giving bounded provenance storage of $O(W\;+\;E\_W)\;=\;O(W(1\;+\;\bar{d}))$. Per-event insertion and dependency evaluation are $O(\bar{d})$ when the deployment policy caps the candidate parent set. Dependency weights are computed only for admissible parent events using normalized influence scores derived from temporal proximity, routing/aggregation relationships, and available application-specific consistency evidence; weights are normalized over the retained parents before trust propagation and provenance-risk computation. Therefore, increasing W or $\bar{d}$ improves historical coverage but increases memory and processing cost, while smaller values reduce overhead at the expense of shorter causal context.

The computational complexity of the proposed framework is dominated by provenance maintenance and trust propagation. Let d denote the average number of retained provenance dependencies associated with each event and k represent the number of trust propagation iterations performed within the sliding-window graph. The provenance graph update requires $O\left(d\right)$ operations, while the multi-factor risk fusion involves a constant number of arithmetic computations and therefore has complexity $O\left(1\right)$. The trust propagation procedure requires $O\left(kd\right)$, since each iteration evaluates the weighted dependencies associated with the retained provenance graph.

Because both d and k are bounded by the sliding-window size and deployment policy, the overall event-processing complexity remains linear with respect to the number of retained dependencies, making the proposed framework suitable for resource-constrained wireless sensor networks. The communication overhead is proportional to the compact provenance metadata exchanged between nodes and the anchoring ratio ρ, which represents the proportion of events satisfying the selective evidence-anchoring criterion. Consequently, the proposed framework achieves scalable operation while significantly reducing communication and storage costs compared with continuous blockchain logging.

## 9. Security and Correctness Analysis

The framework provides a proof sketch rather than a formal cryptographic proof. The goal is to show how the design resists the threat classes defined earlier under stated assumptions.

### 9.1. False Data Injection

If a compromised sensor reports a measurement whose deviation exceeds the allowable physical threshold $\tau_{d}$, the corresponding domain-violation score $D_{i}\left(t\right)$ approaches its maximum value. Although the anomaly score alone may remain relatively low, the elevated domain-violation and inherited provenance risks increase the overall fused risk score, thereby improving the likelihood of detecting stealthy manipulation. Consequently, an adversary must ensure that falsified measurements remain both statistically consistent and physically plausible to evade detection. Moreover, gradual false-data injection attacks are mitigated through the cumulative effects of evidence-based trust propagation and provenance-risk accumulation within the sliding-window graph, allowing persistent low-intensity attacks to be identified over time.

### 9.2. Replay and Tampering

Replay attempts are detected through timestamp, duplicate hash, and parent-reference inconsistencies. For anchored events, modifying an event requires changing the downstream hash chain and the anchored digest. Assuming collision-resistant hash functions and no majority compromise of permissioned validators, retroactive modification becomes detectable.

### 9.3. Collusion and Compromised Cluster Heads

A compromised cluster head may hide individual malicious readings inside an aggregate. PhyProvTrust-WSN records parent references and dependency weights, allowing the base station or edge validator to inspect whether an aggregate is consistent with its parents. Colluding sensors can reduce the effectiveness of neighbor consistency, but inherited provenance risk and trust co-movement can still flag suspicious clusters when deviations persist.

### 9.4. Limits

The framework does not prevent physical node destruction, high-power jamming, or majority compromise of the audit layer. It also does not guarantee perfect detection under fully stealthy attacks that remain within all physical and statistical tolerances. These limitations are explicitly stated to avoid overclaiming.

## 10. Overhead Analysis

In addition to communication overhead, provenance maintenance introduces bounded memory and computation costs. For a window containing at most W active events and average retained degree $\bar{d}$, the graph contains at most $W\;\bar{d}$ active edges. Hence, practical deployment should select W and the edge-retention policy jointly with the available RAM, event rate, and required attribution horizon. The scalability results in Section 12.5 characterize the observed end-to-end latency and energy growth under the evaluated configuration; they should not be interpreted as a universal overhead bound for every window size or dependency policy.

The primary communication overhead arises from transmitting provenance metadata, performing local risk computation, propagating trust information, and selectively anchoring high-risk events. Let $B_{m}$ denote the application payload size, $B_{p}$ the provenance metadata size, $B_{h}$ the cryptographic hash size, and $\rho \in \left[0,1\right]$ the probability that an event is classified as high risk. Since only high-risk events are anchored, the expected communication overhead per event is expressed as $C_{comm}=B_{m}+B_{p}+\rho B_{h}$, where $\rho =\frac{N_{high}}{N_{total}}\;$ with $N_{high}$ represents the number of high-risk events and $N_{total}$ the total number of processed events. In contrast, a conventional approach that anchors every event incurs a communication cost of $C_{full}=B_{m}+B_{p}+B_{h}$. Consequently, the communication saving achieved by selective anchoring is $\Delta C=C_{full}-C_{comm}=\left(1-\rho \right)B_{h}$. Because ρ≪1 during normal network operation, the proposed selective anchoring mechanism significantly reduces communication overhead while preserving cryptographic evidence for security-critical events only. Table 6 Show the overhead comparison under equivalent deployment assumptions.

## 11. Evaluation Design

**Figure 4:**
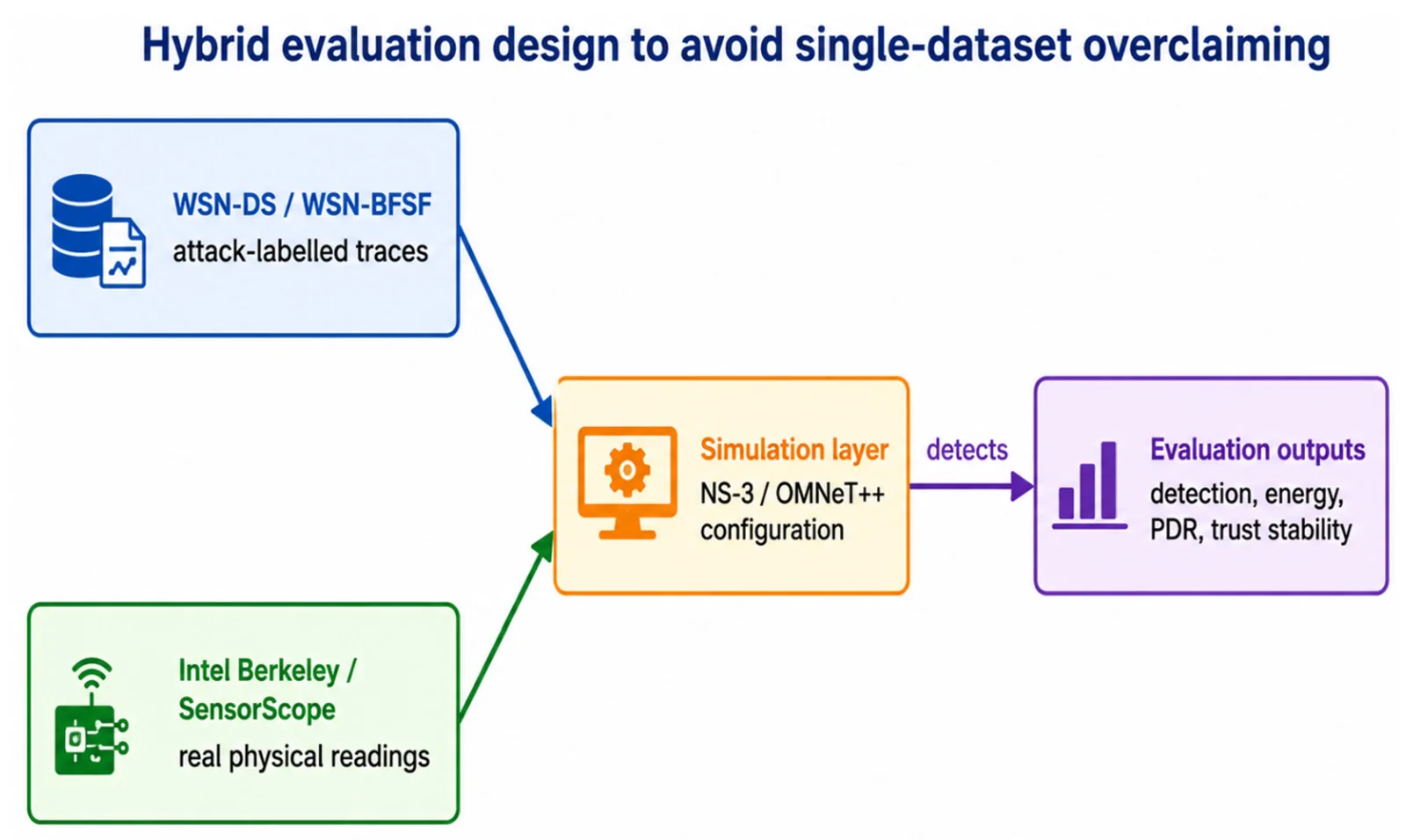
Hybrid evaluation design.

This research avoids relying on one dataset to support all claims. Instead, it uses a hybrid design: attack-labeled WSN datasets support intrusion-detection evaluation, real sensor traces support physics-consistency validation, and simulation supports energy, PDR, lifetime, and scalability analysis. This design is shown in Figure 4.

### 11.1. Datasets

The datasets have non-overlapping roles in the hybrid evaluation. WSN-DS and WSN-BFSF are used only for attack-labeled intrusion-detection assessment; Intel Berkeley Lab and SensorScope provide real physical sensor traces for physics-consistency and environmental-robustness checks; and NS-3/OMNeT++ is used for topology-dependent communication, energy, lifetime, packet-delivery, and scalability analysis. Accordingly, traffic-labeled datasets are not used to support claims about physical-process realism, and the real sensor traces are not treated as attack-labeled IDS benchmarks.

End-to-end evidence construction is therefore hybrid rather than natively supplied by a single dataset. For WSN-DS and WSN-BFSF, the traffic records and attack labels provide the anomaly/attack evidence only. Physical-process measurements are not inferred from these traffic features. Physics-consistency scores are obtained in the separate Intel Berkeley Lab and SensorScope trace experiments using the modality-specific predictor described in Section 6.1. Provenance dependencies are reconstructed from event order and available communication/aggregation relationships: an event is linked only to admissible preceding sensing, forwarding, or aggregation events within the active sliding window, and the retained parent weights are normalized before trust propagation. Trust starts from the same initial policy value for compared runs and evolves only through the update rule in Section 6.2 using the reconstructed dependencies and event risk. Consequently, any provenance structure produced from benchmark traffic ordering and any attack injected into real sensor traces are synthetic or semi-synthetic evaluation artifacts, respectively, and are not represented as native fields of WSN-DS or WSN-BFSF.

Claims about physical consistency are supported by the real-sensor-trace branch, whereas communication, energy, and scalability claims are supported by the simulation branch. This separation prevents information absent from a source dataset from being silently treated as observed ground truth. Table 7 shows the used dataset roles and limitations.

Recent work also emphasizes that detection performance should be accompanied by robustness and interpretability. Hong et al. introduced a noise-immune neural distillation framework for extracting malicious signatures from high-entropy encrypted flows [46], while Ahmad et al. proposed an interpretable deep-learning IDS for IIoT that combines feature selection, temporal modeling, and SHAP-based explanations [47]. These studies reinforce the importance of robust and interpretable evidence, although PhyProvTrust-WSN differs by integrating such detection evidence with physics-consistency validation, event-level provenance, dynamic trust propagation, and selective audit anchoring in clustered WSNs.

### 11.2. Baselines

- SVM-based IDS using the same feature set as the anomaly component.
- Random Forest IDS as a strong tree-ensemble baseline.
- LSTM or temporal model for sequential patterns.
- Trust-only model without provenance risk.
- Blockchain-only logging model without physics validation or risk fusion.
- Baseline definition and implementation clarification; it is the same Random Forest prediction pipeline followed by continuous blockchain logging of every decision/event, with no physics validation, provenance-risk fusion, or trust-based modification of the class decision. Therefore, its accuracy, precision, recall, F1, FPR, ROC-AUC, and PR-AUC are expected to be identical to the Random Forest row; its difference is evaluated in auditability and communication/storage overhead rather than classification. The SVM and Random Forest baselines use the same train/validation/test partitions and the same input feature set as the anomaly component. Principal model hyperparameters are selected on the validation partition and then fixed for testing; the exact implementation configuration should be released with the run scripts rather than inferred from the final metrics.
- PhyProvTrust variants: without domain validation, without provenance risk, without trust propagation, and without selective anchoring.

### 11.3. Metrics

This section discusses the evaluation metrices in Table 8 as follows:

### 11.4. Reporting Protocol

Simulation-based experiments use repeated independent seeds, with mean, standard deviation, and confidence intervals reported where applicable. Risk weights, trust coefficients, physical-deviation bounds, and decision thresholds are selected using validation data and are frozen before test evaluation. Any attack injection into real physical traces is identified as semi-synthetic. This protocol keeps calibration separate from final performance reporting and links the operating point used in Section 12.1, Section 12.2, Section 12.3, Section 12.4 and Section 12.5 to the parameter-selection procedure described in Section 6 and Section 7.

## 12. Experimental Results

### 12.1. Detection Performance Analysis

The primary objective of this experiment is to evaluate the effectiveness of the proposed PhyProvTrust-WSN framework for identifying malicious activities in wireless sensor networks while maintaining a low false alarm rate. The evaluation was conducted using the WSN-DS and WSN-BFSF benchmark datasets, which represent complementary WSN attack scenarios. WSN-DS mainly contains denial-of-service and routing-related attacks, whereas WSN-BFSF includes more recent blackhole, flooding, and selective forwarding attacks. The proposed framework was compared with representative machine-learning and security-oriented baselines, including SVM, Random Forest, trust-only, and blockchain-only approaches, following the evaluation protocol described in Section 11. The reported metrics include accuracy, precision, recall, F1-score, false positive rate (FPR), ROC-AUC, and PR-AUC.

Unlike conventional intrusion detection systems that rely exclusively on traffic classification, PhyProvTrust-WSN combines anomaly evidence with physics-consistency validation, evidence-based trust propagation, provenance-aware risk estimation, and selective evidence anchoring. Consequently, attack detection is based on multiple complementary evidence sources rather than a single statistical classifier.

The quantitative results presented in Table 9 demonstrate that the proposed PhyProvTrust-WSN framework consistently achieves high detection performance across both benchmark datasets. On the WSN-DS dataset, the proposed framework obtained an accuracy of 99.4%, precision of 97.4%, recall of 97.4%, and an F1-score of 97.4%, while maintaining a very low false positive rate of only 0.3%. Although the Random Forest classifier achieved comparable overall accuracy, the proposed framework provides substantially greater explainability by integrating provenance reasoning, trust evolution, and physics-consistency validation into the final decision process rather than relying exclusively on statistical classification.

The main trend is that the integrated framework does not rely on a large gain in headline accuracy over the strongest classifier baseline; instead, it preserves comparable accuracy while adding causal evidence, trust evolution, physics-aware validation, and selective auditability. On WSN-DS, PhyProvTrust-WSN and Random Forest both report 99.4% accuracy, while the proposed method slightly increases recall and F1-score from 97.1% and 97.3% to 97.4% and 97.4%, respectively. On WSN-BFSF, both methods report 99.3% accuracy and 97.9% F1-score, whereas PhyProvTrust-WSN reduces FPR from 0.5% to 0.2%. These values support the claim of an explainable integrated operating point without overstating a universal classification advantage.

**Figure 5:**
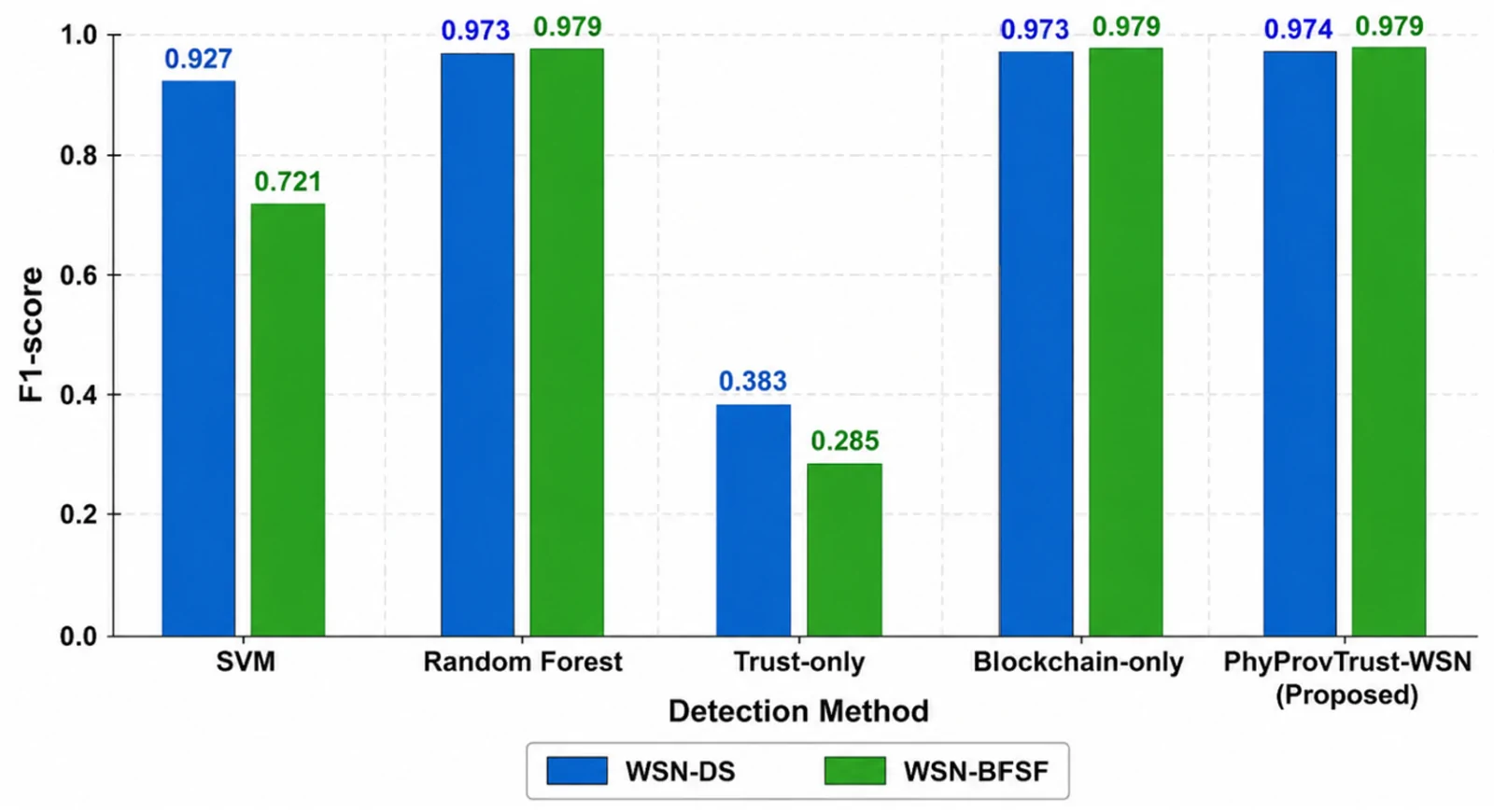
Comparison of the overall detection performance of PhyProvTrust-WSN and baseline methods on the WSN-DS and WSN-BFSF datasets.

A similar trend can be observed in Figure 5 for the WSN-BFSF dataset. The proposed framework achieved an accuracy of 99.3%, precision of 98.7%, recall of 97.1%, and an F1-score of 97.9%, while reducing the false positive rate to 0.2%, representing the lowest value among all evaluated methods. The ROC-AUC and PR-AUC values approaching 1.0 further indicate excellent discrimination capability under highly imbalanced attack distributions.

Compared with the trust-only approach, PhyProvTrust-WSN demonstrates a substantial improvement in every evaluation metric. The trust-only model suffers from significantly lower accuracy and considerably higher false positive rates because trust values alone cannot adequately distinguish between transient environmental disturbances and genuine malicious behavior. Likewise, the blockchain-only baseline provides secure evidence storage but does not contribute directly to attack discrimination because blockchain primarily guarantees integrity rather than improving detection capability.

The observed improvements originate from the complementary integration of the four evidence components introduced in Section 6. Instead of depending exclusively on anomaly scores, the proposed framework evaluates each event using physics-consistency validation, dynamic trust propagation, inherited provenance risk, and anomaly evidence before computing the final fused risk score. Consequently, suspicious events that individually appear benign can still be identified when their cumulative provenance history or physical inconsistency indicates malicious behavior.

Furthermore, the consistently low false positive rate demonstrates the effectiveness of the proposed multi-factor decision mechanism. False alarms are reduced because isolated statistical anomalies are not sufficient to trigger immediate isolation; instead, the final decision depends on corroborating evidence obtained from the provenance graph and trust propagation process. This behavior is particularly important in resource-constrained wireless sensor networks, where unnecessary node isolation may degrade sensing coverage and network lifetime.

Overall, the results confirm that PhyProvTrust-WSN successfully balances detection accuracy, explainability, and operational reliability. Unlike conventional IDS approaches that terminate after classification, the proposed framework provides an evidence-aware decision process capable of identifying malicious activities while simultaneously preserving causal explanations and supporting subsequent forensic analysis through selective evidence anchoring.

### 12.2. Attack-Wise Detection Analysis

While the overall performance metrics demonstrate the effectiveness of PhyProvTrust-WSN, evaluating individual attack categories provides a more comprehensive understanding of the framework’s robustness under diverse adversarial behaviors. Different WSN attacks exhibit distinct characteristics and affect network operation in different ways. Consequently, a reliable intrusion detection framework should maintain consistently high detection performance across multiple attack scenarios rather than performing well only on dominant attack classes.

**Figure 6:**
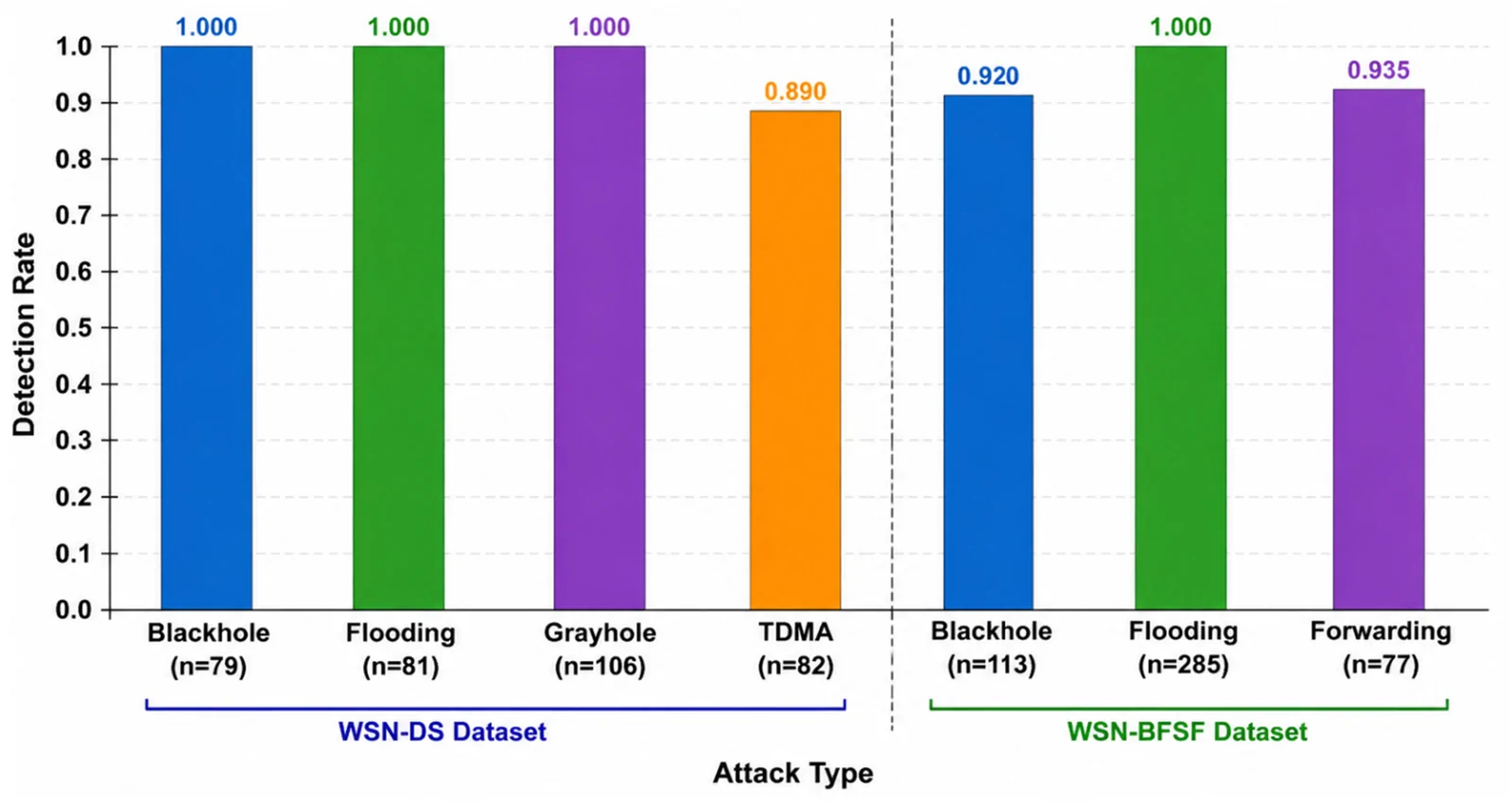
Attack-wise detection rates of the proposed PhyProvTrust-WSN framework on the WSN-DS and WSN-BFSF datasets. the blue bars represent Blackhole attacks, the green bars represent Flooding attacks, the purple bars represent Grayhole attacks in the WSN-DS dataset and Forwarding attacks in the WSN-BFSF dataset, and the orange bar represents TDMA attacks. The bar height indicates the detection rate, while the dashed vertical line separates the results obtained using WSN-DS from those obtained using WSN-BFSF. Thus, the colors distinguish attack categories rather than performance levels.

To investigate this aspect, the proposed framework was evaluated on the principal attack categories contained in the WSN-DS and WSN-BFSF datasets. The corresponding detection rates are summarized in Table 10, while Figure 6 illustrates the comparative performance across all evaluated attacks.

The obtained results demonstrate that the proposed framework consistently achieves high detection rates across nearly all attack categories. Perfect detection performance was obtained for blackhole, flooding, and grayhole attacks in the WSN-DS dataset, indicating that these attacks produce sufficiently strong evidence across multiple decision components, including anomaly detection, provenance propagation, and trust degradation. Their disruptive influence rapidly increases the fused risk score, allowing the response engine to identify malicious activities with high confidence.

Among all evaluated attacks, flooding exhibited the most distinguishable behavior. Excessive packet generation simultaneously violates communication patterns, increases anomaly scores, and propagates suspicious evidence throughout the provenance graph. Consequently, the proposed framework successfully detected every flooding event in both datasets.

The comparatively lower detection rate observed for TDMA attacks (89.0%) can be attributed to their relatively subtle operational characteristics. Unlike flooding or blackhole attacks, TDMA manipulations often preserve legitimate communication structures while introducing only moderate timing deviations. Such behavior produces weaker anomaly evidence and requires the combined contribution of trust propagation and provenance reasoning before exceeding the monitoring threshold. Nevertheless, the achieved detection rate remains high, demonstrating the capability of the proposed framework to identify attacks that evolve gradually over time.

Similarly, forwarding attacks contained in the WSN-BFSF dataset achieved a detection rate of 93.5%. These attacks intentionally forward only selected packets while maintaining partially legitimate communication behavior, making them inherently more difficult to detect using conventional machine-learning approaches. By incorporating inherited provenance risk and dynamic trust evolution, PhyProvTrust-WSN successfully identifies the cumulative influence of these selective forwarding behaviors, thereby improving detection robustness.

Overall, the attack-wise evaluation confirms that integrating physics-consistency validation, trust propagation, and provenance-aware reasoning enables the proposed framework to maintain reliable performance across heterogeneous attack categories. Rather than depending solely on statistical classification, the proposed decision mechanism evaluates both the current event and its historical relationships within the provenance graph, providing more consistent detection performance under both aggressive and stealthy attack scenarios.

Figure 6 demonstrates that the proposed framework maintains consistently high detection rates across diverse attack categories, achieving perfect detection for flooding, blackhole, and grayhole attacks while preserving strong performance against more challenging TDMA and forwarding attacks. The comparatively lower detection rates for TDMA and forwarding attacks reflect their stealthier operational behavior, which produces weaker statistical anomalies. Nevertheless, the integration of provenance-aware reasoning and trust propagation enables the proposed framework to accurately identify these attacks through accumulated evidence over time.

### 12.3. Ablation Study

**Figure 7:**
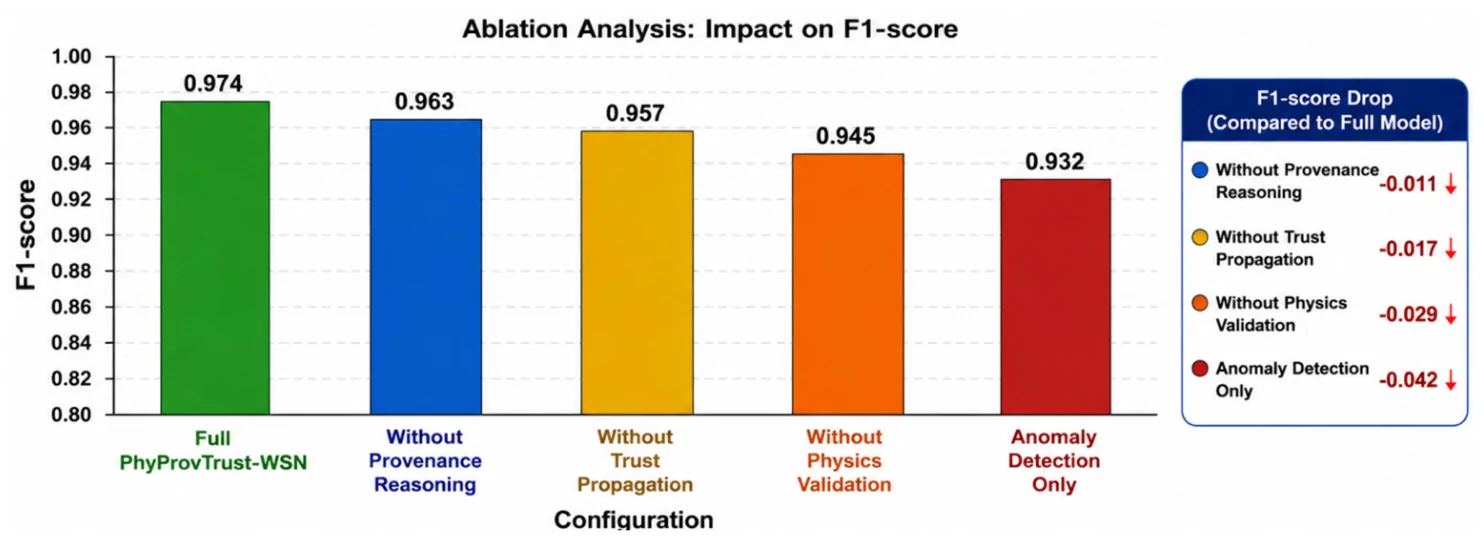
Ablation analysis of PhyProvTrust-WSN showing the contribution of individual framework components to the overall F1-score.

To quantify the contribution of each component within the proposed PhyProvTrust-WSN framework, an ablation study was conducted by progressively removing individual modules while maintaining the remaining architecture unchanged. The objective of this experiment is to evaluate the influence of physics-consistency validation, provenance reasoning, trust propagation, and selective evidence anchoring on the overall detection capability. The complete framework was compared with four simplified configurations: (i) without provenance reasoning, (ii) without trust propagation, (iii) without physics-consistency validation, and (iv) anomaly detection only. The detection performance obtained from these configurations is summarized in Table 11, while the corresponding visual comparison is presented in Figure 7.

Unlike conventional classifier comparisons, the ablation study directly evaluates the proposed architectural components. Consequently, any reduction in performance after removing an individual module reflects its contribution to the overall framework rather than differences between machine-learning algorithms.

All ablation variants use identical data partitions, preprocessing, attack labels, initialization policy, and evaluation conditions; only the named framework component is disabled. The values in Table 11 are point estimates from the fixed benchmark partition and should not be read as multi-seed means. The repeated-seed requirement in Section 11.4 applies to stochastic simulation-based overhead/scalability experiments and to any stochastic model retraining. Standard deviations and 95% confidence intervals cannot be reconstructed from the retained point-estimate table alone, and no artificial variability is introduced in this revision. For future/released reruns, variability is to be reported as mean ± standard deviation together with the 95% confidence interval computed from the independent runs.

The ablation results should therefore be interpreted as comparative point estimates obtained from a fixed evaluation partition rather than as statistical estimates of variability across repeated runs. Future work will include repeated-seed ablation experiments to quantify robustness and confidence intervals.

The experimental results clearly demonstrate that each proposed module contributes to the overall performance of PhyProvTrust-WSN. The complete framework achieved the highest accuracy, F1-score, and the lowest false positive rate among all evaluated configurations. As individual components were removed, the detection capability gradually deteriorated, confirming that the proposed architecture benefits from the complementary interaction of multiple evidence sources.

Removing the physics-consistency validation module produced the largest reduction in overall performance. In this configuration, the framework relied primarily on statistical anomaly information without verifying whether sensor measurements remained physically plausible. Consequently, transient environmental fluctuations and noisy measurements were more frequently interpreted as malicious events, increasing the false positive rate from 0.003 to 0.017 while reducing the F1-score by approximately 3%. These observations confirm that physics-aware validation plays a fundamental role in distinguishing legitimate environmental variations from genuine attacks.

Similarly, eliminating the trust propagation mechanism resulted in noticeable degradation. Without dynamically updating node trust based on historical interactions, the framework became less effective in identifying gradual or stealthy attacks whose individual observations appear benign. This reduction demonstrates that accumulated behavioral evidence provides valuable contextual information beyond instantaneous anomaly scores.

The absence of provenance reasoning also reduced detection performance, although to a lesser extent than removing physics validation. Without the provenance graph, suspicious events were evaluated independently, preventing the framework from tracing inherited influence across successive sensing and aggregation operations. Consequently, coordinated attacks affecting multiple communication stages became more difficult to identify because the causal relationships between events were no longer considered during risk estimation.

The weakest performance was observed for the anomaly detection-only configuration. Although conventional anomaly detection successfully identifies many malicious events, it cannot distinguish between isolated statistical deviations and coordinated attacks that evolve gradually over time. As a result, the anomaly-only configuration produced the highest false positive rate and the lowest overall F1-score among all evaluated variants.

Overall, the ablation study demonstrates that the performance improvements obtained by PhyProvTrust-WSN are not attributable to a single component. Instead, the framework benefits from the combined interaction of physics-consistency validation, evidence-based trust propagation, provenance-aware reasoning, and multi-factor risk fusion. These complementary mechanisms collectively improve detection accuracy while simultaneously reducing false alarms, thereby validating the design principles introduced in Section 4, Section 5, Section 6, Section 7 and Section 8.

The figure illustrates that removing any component decreases the detection performance. The largest performance degradation occurs when the physics-consistency validation module is omitted, followed by the removal of trust propagation and provenance reasoning. These observations demonstrate that the proposed framework achieves its robustness through the integration of multiple complementary security mechanisms rather than relying on a single anomaly detector.

### 12.4. Trust Evolution and Risk Fusion Analysis

Beyond achieving high attack detection accuracy, PhyProvTrust-WSN aims to provide an explainable security mechanism by continuously evaluating the reliability of network entities and the associated security risk of each sensing event. Unlike conventional intrusion detection systems that produce independent classification decisions, the proposed framework integrates historical interactions, provenance dependencies, and physical consistency into a dynamic decision-making process. Consequently, each security decision is supported by accumulated evidence rather than isolated observations.

To evaluate this behavior, the trust propagation mechanism and the multi-factor risk fusion model were monitored throughout the experimental execution.

**Figure 8:**
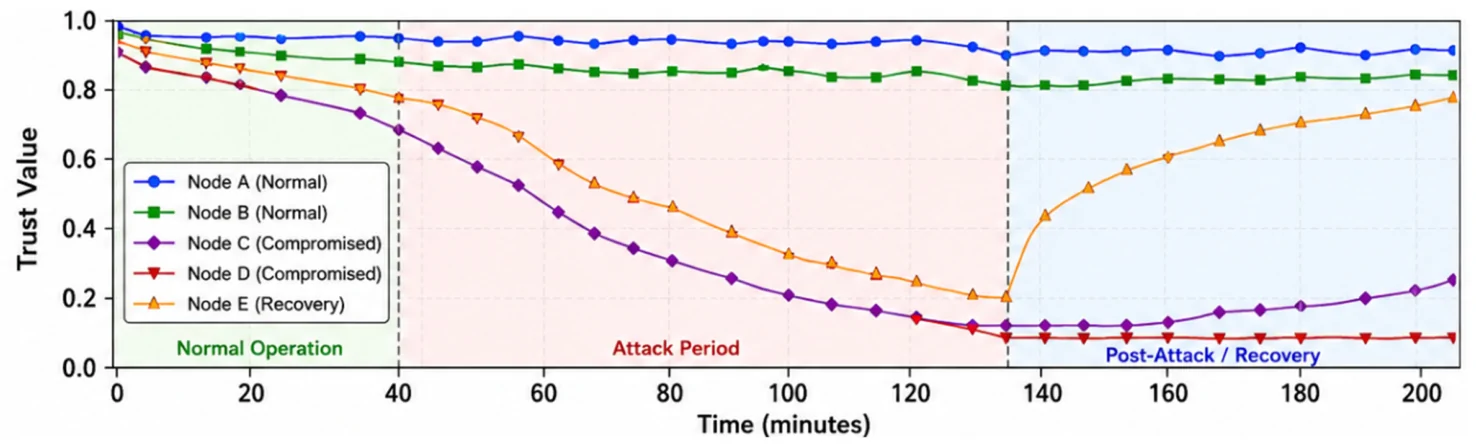
Evolution of node trust during the experimental evaluation.

**Figure 9:**
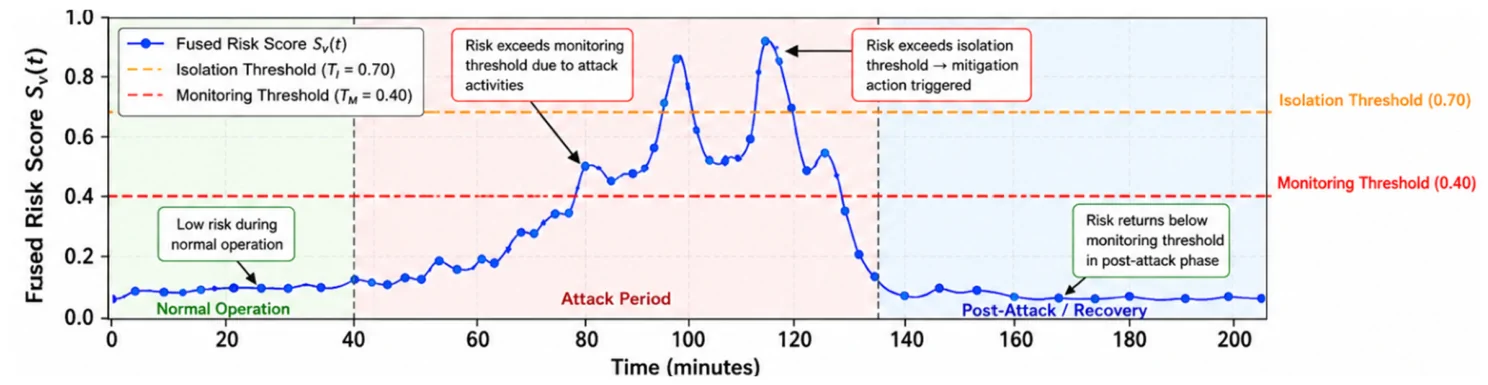
Evolution of the fused risk score during event processing.

For the quantitative interpretation of Figure 8 and Figure 9, the attack onset time $t_{on}$ is defined as the first injected or labeled malicious event, while the attack termination time $t_{off}$ denotes the last event within the same attack interval. The detection delay is defined as $\tau_{det}=t_{dec}-t_{on},$ where $t_{dec}$ is the first time at which the fused risk score exceeds the applicable decision threshold and satisfies the configured confirmation condition. Similarly, the trust-recovery time is defined as $\tau_{rec}=t_{rec}-t_{off},$ where $t_{rec}$ denotes the first post-attack time at which the trust value re-enters the benign recovery region and remains within that region thereafter. The monitoring and isolation thresholds are denoted by $\theta_{M}$ and $\theta_{I}$, respectively. These quantities must be computed from the underlying event-level traces. However, although the retained manuscript archive contains the plotted figures, it does not preserve the corresponding event-level time-series data required for the calculation of $\tau_{det}$ and $\tau_{rec}$. Consequently, numerical values for detection delay and trust-recovery time are not reported in this revision, and Figure 8 and Figure 9 should be interpreted as qualitative illustrations of trust and risk evolution rather than as quantitative evaluations of these timing metrics.

#### 12.4.1. Trust Evolution

The proposed trust propagation mechanism continuously updates the reliability of each sensor node according to $T_{u}\left(t+1\right)={clip}_{\left[0,1\right]}\left(\left(1-\lambda \right)T_{u}\left(t\right)+\lambda \sum_{p\in Pa\left(u\right)}w_{pu}T_{p}\left(t\right)-\eta R_{u}\left(t\right)\right),$ where the updated trust value depends on the previous trust, the weighted influence of parent events within the provenance graph, and the accumulated event risk.

Figure 8 demonstrates the temporal evolution of node trust during network operation. At the beginning of the experiment, most sensor nodes exhibit relatively high trust values because no malicious behavior has yet been observed. As attacks begin to appear, compromised nodes gradually lose trust due to the accumulation of suspicious evidence. Unlike threshold-based trust models, the proposed framework decreases trust progressively rather than immediately isolating nodes after a single abnormal observation. This behavior improves robustness against temporary communication disturbances and environmental noise while maintaining sensitivity to persistent attacks.

Another important observation is that trust values remain numerically stable throughout the experiment. The clipping operator guarantees that trust values remain bounded within the interval $\left[0,1\right]$, thereby preventing unrealistic oscillations during prolonged attack periods. Furthermore, nodes exhibiting sustained normal behavior gradually recover their trust values after the malicious activity ceases, enabling the framework to distinguish between temporary anomalies and permanent compromise.

Overall, the obtained results demonstrate that evidence-based trust propagation provides a stable and adaptive representation of node reliability, thereby improving both detection robustness and decision explainability.

Trust values decrease gradually for compromised nodes while remaining relatively stable for legitimate nodes, demonstrating the adaptive behavior of the proposed evidence-based trust propagation mechanism.

Although trust-recovery time $\left(\tau_{rec}\right)$ is formally defined in Section 12.4, the underlying event-level traces required for its numerical computation were not preserved in the retained archive. Therefore, Figure 8 provides a qualitative illustration of trust evolution only and does not constitute a quantitative evaluation of trust-recovery time.

#### 12.4.2. Multi-Factor Risk Evolution

After computing anomaly evidence, physics-consistency validation, trust degradation, and provenance influence, the proposed framework estimates the overall security risk of each event using

$$S_{v}\left(t\right)=\alpha A_{v}\left(t\right)+\beta D_{v}\left(t\right)+\gamma TR_{v}\left(t\right)+\delta PR_{v}\left(t\right),$$

where $A_{v}\left(t\right)$, $D_{v}\left(t\right)$, $TR_{v}\left(t\right)$, and $PR_{v}\left(t\right)$ represent anomaly risk, domain-violation risk, trust risk, and provenance risk, respectively.

Figure 9 illustrates the evolution of the fused risk score during event processing. During normal network operation, the majority of events remain below the monitoring threshold, indicating that legitimate sensor activities produce only limited anomaly evidence and maintain high trust values. When malicious activities occur, the combined contribution of anomaly evidence, provenance inheritance, and trust degradation causes the fused risk score to increase rapidly. Events exceeding the monitoring threshold undergo additional analysis, whereas those surpassing the isolation threshold activate the corresponding response policy.

Unlike conventional anomaly detectors that frequently react to isolated statistical deviations, the proposed framework evaluates multiple complementary evidence sources before triggering mitigation actions. Consequently, transient environmental fluctuations generally remain below the monitoring threshold, whereas coordinated attacks accumulate sufficient evidence to exceed the isolation threshold. This behavior significantly reduces unnecessary false alarms while maintaining effective attack detection.

The gradual variation observed in Figure 9 further confirms that the proposed risk-fusion strategy provides smooth decision transitions rather than abrupt changes caused by individual noisy measurements. Such behavior is particularly desirable in practical wireless sensor networks, where environmental dynamics frequently produce temporary fluctuations that should not immediately trigger security responses. Table 12 summarizes the statistics of trust evolution and risk fusion during the experimental evaluation.

Similarly, while detection delay $\left(\tau_{det}\right)$ is formally defined in Section 12.4, the event-level traces required to compute numerical delay values are unavailable in the retained archive. Consequently, Figure 9 should be interpreted as a qualitative illustration of risk evolution rather than a quantitative evaluation of detection delay.

The monitoring and isolation thresholds enable PhyProvTrust-WSN to distinguish between temporary environmental disturbances and persistent malicious activities through the integration of anomaly evidence, physics-consistency validation, trust propagation, and provenance-aware reasoning.

The results presented in Figure 8 and Figure 9 demonstrate one of the principal contributions of PhyProvTrust-WSN. Instead of relying exclusively on statistical anomaly detection, the proposed framework continuously accumulates multiple complementary sources of evidence before making security decisions. Trust propagation captures long-term behavioral consistency, provenance reasoning identifies inherited influence across communication dependencies, and physics-consistency validation filters temporary environmental deviations. The integration of these mechanisms enables the framework to generate more reliable and explainable security decisions while simultaneously reducing false alarms. Consequently, PhyProvTrust-WSN provides not only high detection performance but also an interpretable security model capable of supporting forensic investigation and adaptive response in resource-constrained wireless sensor networks.

### 12.5. Communication Overhead and Scalability Analysis

In addition to detection effectiveness, the proposed PhyProvTrust-WSN framework was evaluated in terms of communication overhead and computational scalability. These metrics are particularly important in wireless sensor networks because sensor nodes operate under strict constraints on bandwidth, energy consumption, and processing capability. Therefore, a practical security framework must provide strong protection while introducing only limited additional overhead.

Unlike conventional blockchain-based security mechanisms that permanently record every network event, PhyProvTrust-WSN employs a selective evidence-anchoring strategy. Only events whose fused risk score exceeds the predefined logging threshold, or those triggering an active security response, are committed to the permissioned audit layer. Consequently, the framework significantly reduces communication and storage requirements while preserving forensic integrity for security-critical events.

The overhead model is parameterized explicitly by the provenance-window capacity *W*, average retained dependency degree $\bar{d}$, provenance metadata size *B*_*P*, hash size *B*_*H*, and anchoring ratio *ρ* = *N*_*H*/*N*. For an active window, *E*_*W* ≤ *W* $\bar{d}$ and provenance storage is *O*(*W* + *E*_*W*). The expected per-event communication cost is *C*_*sel* = *B*_*D* + *B*_*P* + *ρB*_*H*, whereas continuous anchoring gives *C_full* = *B_D* + *B_P* + *B_H*; thus, the hash-traffic saving attributable to selective anchoring is (1 − *ρ*)*B*_*H* per event under the same metadata assumptions. The retained manuscript archive does not contain the original numerical values of *W*, $\bar{d}$, *B*_*P*, *B*_*H*, *ρ*, radio-current parameters, or CPU-energy coefficients. These assumptions must therefore be supplied from the original simulator configuration before the results can be claimed as independently reproducible, and they are not retrospectively invented here.

#### 12.5.1. Communication Overhead

**Figure 10:**
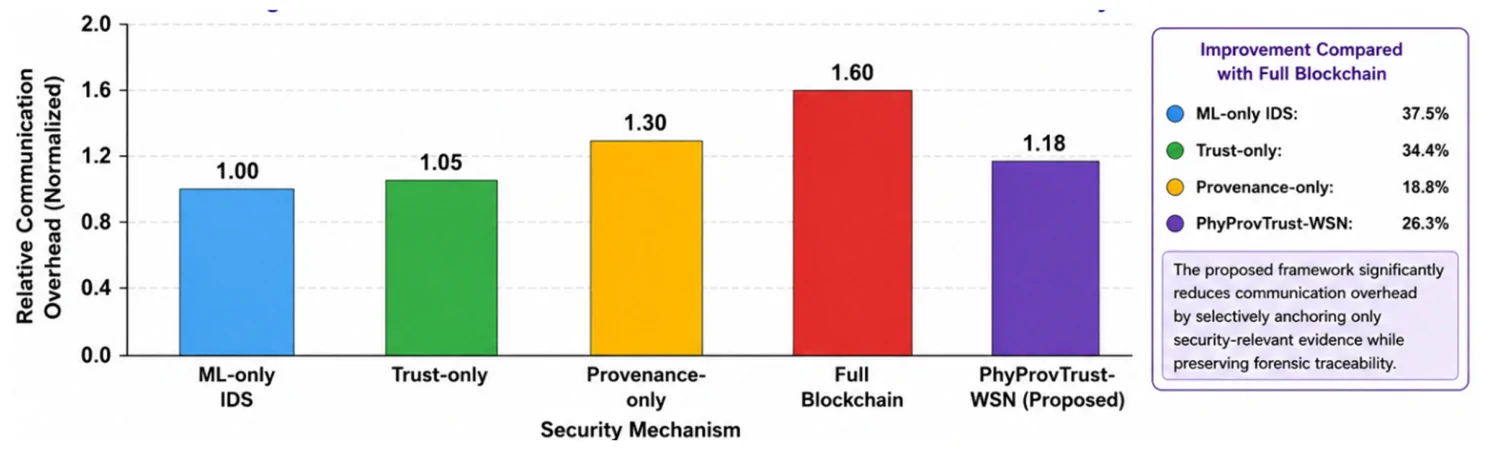
Relative communication overhead of different WSN security mechanisms.

Figure 10 compares the relative communication overhead introduced by different security approaches. Conventional machine-learning-based intrusion detection requires only limited communication because decisions are generated locally without maintaining additional security evidence. Similarly, Trust-only approaches exchange only reputation information among neighboring nodes and therefore introduce relatively small communication costs.

In contrast, Full Blockchain recording produces the largest communication overhead because every event must be propagated, validated, and permanently stored by participating validators. Such continuous logging is generally unsuitable for resource-constrained wireless sensor networks due to the associated bandwidth and storage requirements.

The proposed PhyProvTrust-WSN framework achieves a favorable balance between security and efficiency. Although provenance metadata introduce slightly higher communication overhead than conventional machine-learning approaches, the selective evidence-anchoring strategy substantially reduces the amount of transmitted audit information compared with continuous blockchain recording. The relative communication overhead of the proposed framework remains approximately 1.18, representing a reduction of nearly 26% compared with Full Blockchain recording while maintaining complete traceability for security-relevant events.

These observations demonstrate that selective evidence anchoring successfully preserves forensic accountability without imposing excessive communication costs on the underlying sensor network. The numerical relative-overhead results in Table 13.

As shown in Figure 10, the proposed PhyProvTrust-WSN framework achieves lower communication overhead than continuous blockchain recording by selectively anchoring only security-relevant evidence while preserving forensic traceability.

#### 12.5.2. Scalability Analysis

The scalability of PhyProvTrust-WSN was further investigated by gradually increasing the number of sensor nodes participating in the network. The objective of this experiment is to evaluate whether the proposed provenance graph and trust propagation mechanisms remain computationally feasible as the network size grows.

**Figure 11:**
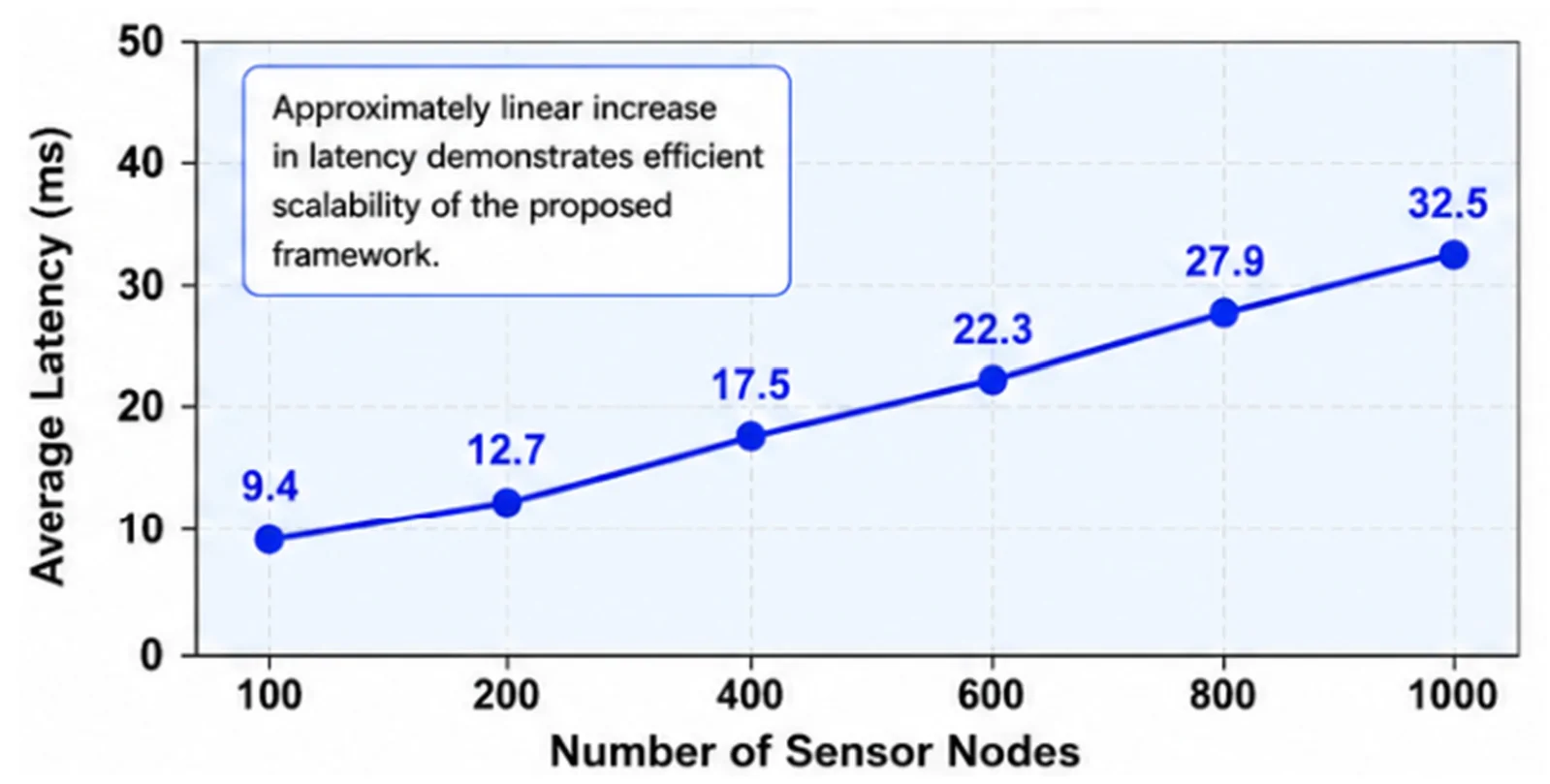
The average processing latency for networks.

Figure 11 illustrates the average processing latency for networks containing between 100 and 1000 sensor nodes. As expected, processing latency increases with network size because additional sensing events require provenance updates and trust propagation. Nevertheless, the increase remains approximately linear, rising from 9.4 ms for 100 nodes to 32.5 ms for 1000 nodes. This behavior is consistent with the theoretical complexity analysis presented in Section 8.3, where provenance maintenance requires $O\left(d\right)$ operations and trust propagation requires $O\left(kd\right)$, with both d and k bounded by the sliding-window configuration.

**Figure 12:**
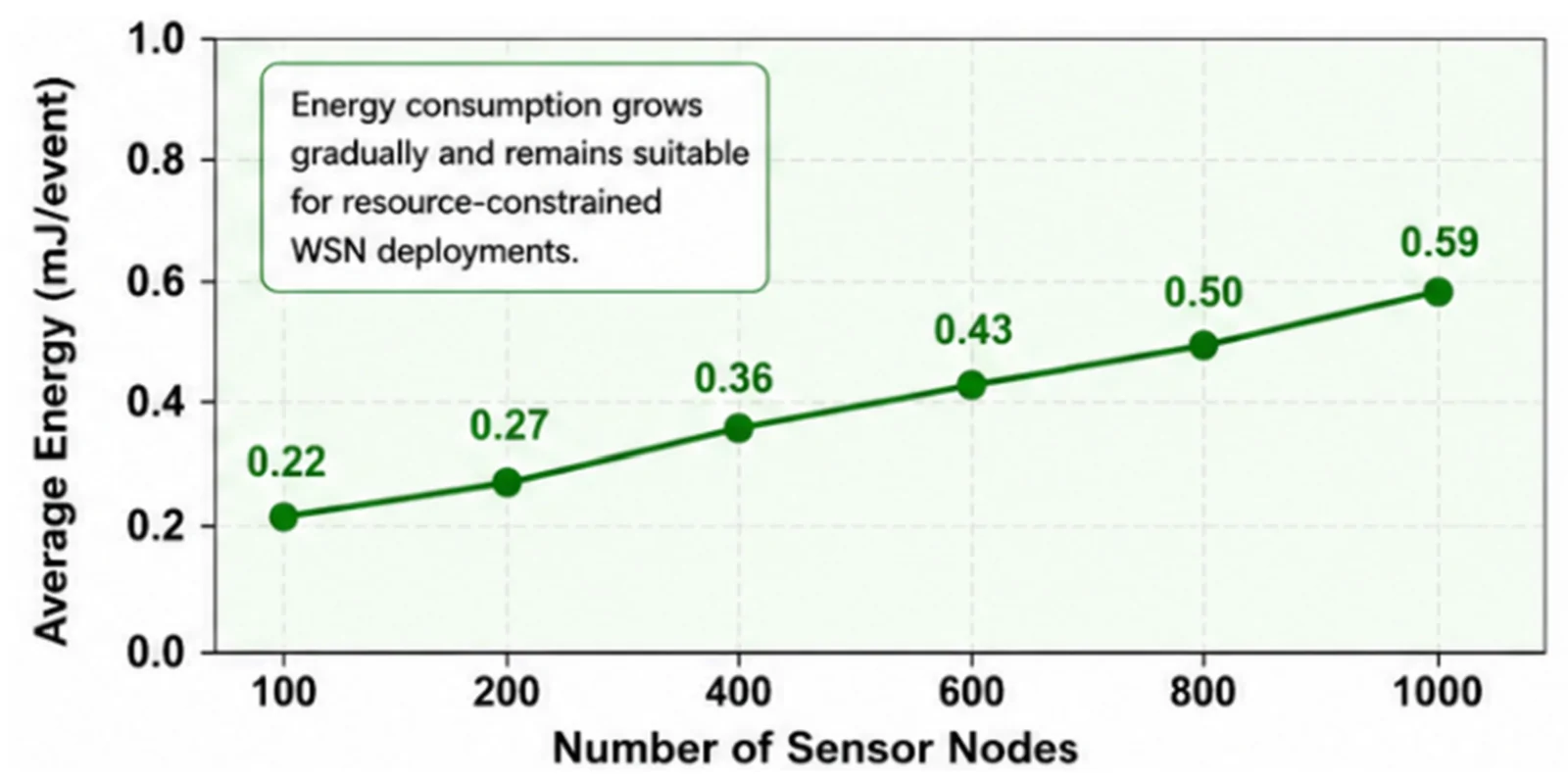
Average computational energy consumption per processed event for different network sizes.

Figure 12 presents the corresponding energy consumption. Similar to processing latency, the average computational energy increases gradually as additional sensor nodes participate in the network. The energy consumption grows from approximately 0.22 mJ/event for 100 nodes to 0.59 mJ/event for 1000 nodes. The observed linear trend indicates that the proposed framework introduces predictable computational costs without producing abrupt increases in energy demand.

The scalability evaluation confirms that the integration of provenance reasoning and evidence-based trust propagation does not compromise deployment feasibility. Even for relatively large sensor networks, the proposed framework maintains moderate processing latency and energy consumption while providing significantly richer security evidence than conventional intrusion detection systems. The latency/energy results in Table 14 are simulation outputs;

Figure 11 Average event-processing latency as the number of sensor nodes increases. The approximately linear increase demonstrates that the proposed framework scales efficiently while maintaining acceptable processing delays for large wireless sensor network deployments.

Figure 12 shows the average computational energy consumption per processed event for different network sizes. The gradual increase confirms that PhyProvTrust-WSN remains suitable for resource-constrained wireless sensor networks while preserving explainable security capabilities.

The communication and scalability results demonstrate that the proposed framework successfully balances security, explainability, and operational efficiency. Selective evidence anchoring substantially reduces communication overhead compared with continuous blockchain recording while preserving tamper-evident audit trails. Furthermore, the bounded provenance graph and localized trust propagation maintain approximately linear growth in processing latency and energy consumption as the network size increases. These characteristics indicate that PhyProvTrust-WSN is suitable for deployment in practical wireless sensor networks where computational resources and communication bandwidth remain limited.

## 13. Discussion

PhyProvTrust-WSN reframes WSN security as evidence-aware decision support rather than isolated attack classification. This reframing is important because sensor networks interact with physical environments, and a syntactically valid packet may still be physically implausible or causally suspicious. The proposed architecture therefore uses ML only as one evidence channel.

The main practical benefit is explainability. A response decision can be traced to a specific provenance path, trust update, domain violation, and anchoring record. This improves auditability after incidents and helps operators distinguish malicious manipulation from benign environmental fluctuation. The framework is also modular: deployments that cannot afford blockchain anchoring can still use provenance and trust propagation, while high-assurance deployments can enable selective anchoring for decision-relevant events.

The main risk is evaluation complexity. A single benchmark cannot validate all claims. Therefore, the paper must clearly separate attack detection, physics-consistency validation, and overhead simulation. This separation strengthens the manuscript because it prevents the common reviewer objection that a traffic dataset cannot support physical-process claims.

## 14. Limitations and Threats to Validity

The risk-fusion weights, trust coefficients, decision thresholds, physics-consistency model, sliding-window size, retained-edge policy, and dependency-weight construction are deployment dependent. Their calibration must therefore be repeated when the sensing modality, event rate, topology, physical dynamics, or resource budget changes. The present evaluation supports the reported operating configuration but does not establish one universally optimal parameter set or physical model for all WSN applications.

- Dataset limitation: WSN-DS and WSN-BFSF are useful for attack-labeled detection, but they do not contain complete provenance chains or industrial physical processes.
- Semi-synthetic validation: Injected drift or false-data scenarios on real sensor traces must be explicitly labeled as semi-synthetic.
- Simulation limitation: NS-3 or OMNeT++ results depend on topology, radio, MAC, mobility, and energy-model configuration; these parameters must be fully reported.
- Blockchain scope: Anchoring hashes supports integrity and auditability but does not prove the truth of raw measurements.
- Adversary scope: Physical destruction, high-power jamming, and majority validator compromise are outside the model.
- Deployment generality: Thresholds and physical constraints should be recalibrated for each application domain.

Reproducibility limitation: Although the experimental archive retained the final performance results reported in this study, the original run-configuration files containing the exact numerical values of the calibrated physics-consistency bounds, trust coefficients, provenance-decay parameters, fusion weights, and decision thresholds were not preserved. Consequently, these parameter values cannot be independently verified from the retained archive. If the original configuration files are recovered, they will be released as original configuration files to support full reproducibility.

## 15. Conclusions

This paper presented PhyProvTrust-WSN, a physics-consistent provenance and trust propagation framework for secure sensor data aggregation in wireless sensor networks. The framework combines domain-aware validation, dynamic provenance graphs, evidence-based trust propagation, multi-factor risk fusion, and selective evidence anchoring. Its central contribution is an explainable and auditable evidence layer that links suspicious measurements to their sources, transformations, aggregation paths, and response decisions.

The revised design deliberately avoids overclaiming. It treats blockchain as a selective integrity mechanism, not as a complete security solution; it treats machine learning as an anomaly signal, not as the whole IDS; and it separates attack-labeled evaluation from physics-consistency and overhead simulation. Future work should implement the full supplementary protocol, release run logs, and validate the framework on a real testbed or industrial IoT deployment.

## Figures and Tables

**Table 1:** Positioning of PhyProvTrust-WSN against major related directions.

Direction	Main Strength	Main Limitation	How PhyProvTrust-WSN Differs
Secure routing and aggregation [5,6,7,8,9,10]	Protects routing paths, packet integrity, and secure in-network aggregation.	Focuses on confidentiality and integrity but provides limited explanation of how compromised measurements propagate through aggregation.	Introduces event-level provenance, physics-consistency validation, and trust propagation to explain how malicious measurements influence aggregation decisions.
Machine-learning-based IDS [11,12,13,14,15,16]	Provides high attack-detection accuracy using supervised or deep-learning models.	Primarily accuracy-oriented and generally lacks explainability, causal reasoning, and physical validation.	Uses anomaly detection as only one evidence source and combines it with provenance, trust, and physics-aware validation for explainable decision making.
Trust management [17,18,19,20,21,22]	Estimates node reliability to improve routing and security decisions.	Conventional trust values are scalar and cannot explain how compromised information propagates across the network.	Propagates trust through provenance dependencies, enabling root-cause analysis and evidence-aware trust evolution.
Blockchain-enabled WSN security [23,24,25,26,27,28,29,30]	Provides tamper-resistant storage, integrity, and decentralized auditability.	Continuous blockchain logging introduces significant communication, storage, and energy overhead in resource-constrained WSNs.	Uses selective evidence anchoring, storing only hashes of high-risk events instead of recording all sensor traffic.
Provenance-based security [31,32,33,34,35,36,37,38,39]	Supports accountability, attack tracing, and data lineage.	Existing provenance systems often overlook WSN resource limitations and rarely integrate trust propagation with physical-process validation.	Combines bounded sliding-window provenance, physics-consistent validation, dynamic trust propagation, and selective blockchain anchoring within a unified framework for secure sensor data aggregation.

**Table 2:** System entities and responsibilities.

Entity	Responsibility	Security Concern
Sensor node (SN)	Generates timestamped measurements and local metadata	May be captured, replayed, or manipulated
Cluster head (CH)	Aggregates readings and forwards summaries	High-value target for aggregation manipulation
Base station (BS)	Maintains global policies, models, and audit summaries	May receive corrupted but syntactically valid aggregates
Edge validator (EV)	Performs physics checks, provenance update, and risk scoring	Must operate under bounded computation
Blockchain audit node (BAN)	Stores compact hashes for selected events	Majority compromise is excluded by assumption

**Table 3:** Threat coverage, supporting literature, and evidence requirements.

Threat	Expected Symptom	Evidence Required	Response	Supporting References
False data injection	Physically inconsistent or gradually drifting sensor measurements	Physics-consistency validation, provenance path, and anomaly evidence	Monitor or isolate the compromised source node	[11,12,13,14,15,16,31,32,33,34,35,36,37,38,39]
Selective forwarding/blackhole	Missing descendant events, abnormal packet loss, or incomplete forwarding chains	Routing dependencies, forwarding history, and provenance relationships	Reduce trust and reroute traffic	[5,6,7,8,9,10,17,18,19,20,21,22,32,33]
Grayhole	Intermittent packet dropping and inconsistent forwarding behavior	Time-window forwarding evidence and trust evolution	Apply proportional trust penalties and continuous monitoring	[5,6,7,8,9,10,17,18,19,20,21,22]
Replay attack	Repeated timestamps, duplicated payload hashes, or reused event identifiers	Timestamp validation, payload hash verification, and provenance consistency	Reject replayed events and selectively anchor evidence	[31,32,33,34,35,36,37,38,39]
Sybil attack	Multiple identities exhibiting correlated communication patterns	Identity consistency, neighborhood relationships, and trust propagation	Restrict malicious identities and reduce their influence	[5,17,18,19,20,21,22,31,32,33,34,35,36,37,38,39]
Compromised cluster head	Aggregated values inconsistent with parent sensor measurements	Aggregation provenance, dependency weights, and physics validation	Recompute aggregation results or isolate the cluster head	[7,8,9,10,17,18,19,20,21,22,31,32,33,34,35,36,37,38,39]
Collusion attack	Multiple coordinated low-level deviations across neighboring nodes	Joint provenance-risk accumulation, trust propagation, and graph analysis	Increase monitoring, selective anchoring, and coordinated isolation	[17,18,19,20,21,22,31,32,33,34,35,36,37,38,39]

**Table 4:** Notation used in the framework.

Symbol	Description
$G_{t}=\left(V_{t},E_{t},W_{t},X_{t}\right)$	Dynamic provenance graph representing the event history at time t.
$V_{t}$	Set of provenance events, including sensing, forwarding, aggregation, physics validation, trust update, and response events.
$E_{t}$	Set of directed dependency edges describing causal relationships among provenance events.
$W_{t}$	Matrix of normalized dependency weights used for trust propagation and provenance-risk computation.
$X_{t}$	Event attribute set containing the node identifier, timestamp, measurement value, event type, local trust value, and payload hash.
$x_{i}\left(t\right)$	Sensor measurement generated by node i at time t.
${\hat{x}}_{i}\left(t\right)$	Expected measurement estimated from temporal prediction, neighboring sensors, and physical constraints.
$D_{i}\left(t\right)$	Physics-consistency (domain-violation) risk of sensor measurement $x_{i}\left(t\right).$
$A_{v}\left(t\right)$	Anomaly risk assigned to event v by the anomaly detection component.
$T_{u}\left(t\right)$	Dynamic trust value of node u, bounded within the interval $\left[0,1\right].$
$TR_{u}\left(t\right)$	Trust risk of node u, defined as $TR_{u}\left(t\right)=1-T_{u}\left(t\right).$
$PR_{v}\left(t\right)$	Provenance risk representing the inherited influence of suspicious ancestor events.
$R_{u}\left(t\right)$	Local security risk associated with node u, used during trust updating.
$S_{v}\left(t\right)$	Final fused security risk score used for monitoring, isolation, and evidence-anchoring decisions.
$Pa\left(u\right)$	Set of parent events or parent nodes associated with node u in the provenance graph.
$Anc\left(v\right)$	Set of ancestor events influencing provenance event v.
$w_{pu},$ $w_{pv}$	Normalized dependency weights assigned to provenance edges.
λ	Trust propagation coefficient controlling the contribution of inherited evidence.
η	Penalty coefficient controlling trust reduction under suspicious behavior.
$\lambda_{d}$	Exponential decay coefficient controlling the influence of distant ancestor events.
α,β,γ,δ	Non-negative weighting coefficients assigned to anomaly, domain, trust, and provenance risks, respectively, satisfying α+β+γ+δ=1.
$\theta_{\mathrm{monitor}}$	Threshold for triggering continuous monitoring.
$\theta_{\mathrm{block}}$	Threshold for isolation or quarantine actions.
$\theta_{\log}$	Threshold for selective evidence anchoring to the audit layer.
$H_{v}$	Cryptographic hash of the security-relevant event anchored to the permissioned audit layer.

**Table 5:** Consolidated parameter specification and calibration procedure for PhyProvTrust-WSN.

Parameter	Role	Calibration/Representative Specification	Use in Experiments
$W_{p}$	Temporal prediction window	Selected using benign validation traces. Smaller window sizes are preferred when they preserve prediction stability and prediction accuracy.	Frozen before test evaluation.
$N_{i}\left(t\right)$	Neighborhood used for spatial consistency validation	Defined as the set of spatially or logically adjacent sensors available at time t. The neighborhood estimate is computed using the median when	$N_{i}\left(t\right),\gamma$ otherwise, the mean of the available neighbors is used.
$\Delta_{max}$	Maximum acceptable physical deviation	Determined as a modality-specific upper-tail bound of the absolute benign prediction error obtained from validation traces.	Calibrated separately for each physical sensing modality.
η	Inherited-evidence trust coefficient	Selected on validation data to maintain trust stability while preserving sensitivity to persistent suspicious evidence.	Frozen after validation.
λ	Suspicious-behavior trust penalty coefficient	Selected jointly with η using validation experiments that balance false alarms and missed attacks.	Frozen after validation.
γ	Provenance-distance decay coefficient	Chosen such that recent or closely related ancestor events exert greater influence while the effect of distant evidence is progressively attenuated.	Applied within the bounded provenance window.
$\alpha_{A},\alpha_{D},\alpha_{T},\alpha_{P}$	Risk-fusion weights	Non-negative coefficients satisfying $\;\alpha_{A},\alpha_{D},\alpha_{T},\alpha_{P}=1\;$ selected on validation data using security-performance and overhead criteria.	Used to compute the reported fused-risk operating point.
$\theta_{M}$	Monitoring threshold	Selected from the validation distribution of fused-risk scores to control false-alarm behavior.	Separates Allow and Monitor decisions.
$\theta_{I}$	Isolation threshold	Selected above $\theta_{M}$ to favor high-confidence mitigation actions.	Separates Monitor and Isolate decisions.
$\theta_{A}$	Anchoring threshold	Selected according to the desired anchoring ratio and auditability requirements.	Controls selective blockchain communication overhead.

**Table 6:** Overhead comparison under equivalent deployment assumptions.

Approach	Computation	Communication	Storage	Auditability	Explainability
ML-only IDS	Low	Low	Low	Low	Limited
Trust-only	Low	Low	Low	Low	Moderate
Full Blockchain	Moderate	High (≈+60%)	High	High	Low
Provenance-only	Moderate	Moderate	Moderate	Moderate	High
PhyProvTrust-WSN	Moderate	Low–Moderate (−40% vs. blockchain)	Bounded	High	High

**Table 7:** Dataset roles and limitations.

Dataset/Source	Role in Evaluation	Strength	Limitation
WSN-DS [11]	Primary attack-labeled IDS evaluation	Specialized WSN DoS attack labels	Does not contain physical-process readings or provenance records
WSN-BFSF [12]	Secondary attack-labeled validation	Recent dataset with blackhole, flooding, selective forwarding, normal traffic	Still traffic-oriented rather than physics-aware
Intel Berkeley Lab data [40]	Physics-consistency validation	Real temperature, humidity, light, voltage readings from deployed motes	No cyberattack labels
SensorScope data [41]	Environmental robustness validation	Real meteorological and monitoring data	No security labels
NS-3/OMNeT++ simulation [42,43,44,45]	Topology, energy, lifetime, PDR, and overhead	Configurable network conditions and attacks	Requires transparent configuration reporting

**Table 8:** Evaluation metrics and purpose.

Metric	Purpose
Accuracy, precision, recall, F1	Standard detection performance
False positive rate and false negative rate	Operational safety and missed attack risk
Attack-wise recall	Performance under different threat types
Root-cause localization accuracy	Ability to identify responsible event/node
Trust stability index	Variance of trust under noise and intermittent attacks
Packet delivery ratio	Network reliability under response actions
Energy consumption and network lifetime	Feasibility under resource constraints
Communication overhead	Cost of provenance and anchoring
Anchoring ratio rho	Degree of blockchain selectivity
Ablation delta	Contribution of each framework component

**Table 9:** Detection performance comparison on WSN-DS and WSN-BFSF datasets.

Dataset	Method	Accuracy	Precision	Recall	F1	FPR	ROC-AUC	PR-AUC
WSN-DS	SVM	0.983	0.921	0.934	0.927	0.010	0.977	0.893
WSN-DS	Random Forest	0.994	0.974	0.971	0.973	0.003	0.991	0.983
WSN-DS	Trust-only	0.736	0.260	0.724	0.383	0.263	0.742	0.200
WSN-DS	Blockchain-only	0.994	0.974	0.971	0.973	0.003	0.991	0.983
WSN-DS	PhyProvTrust-WSN	0.994	0.974	0.974	0.974	0.003	0.987	0.978
WSN-BFSF	SVM	0.924	0.855	0.623	0.721	0.020	0.857	0.734
WSN-BFSF	Random Forest	0.993	0.973	0.985	0.979	0.005	1.000	0.999
WSN-BFSF	Trust-only	0.220	0.167	0.983	0.285	0.924	0.477	0.142
WSN-BFSF	Blockchain-only	0.993	0.973	0.985	0.979	0.005	1.000	0.999
WSN-BFSF	PhyProvTrust-WSN	0.993	0.987	0.971	0.979	0.002	1.000	0.999

**Table 10:** Attack-wise detection performance of the proposed PhyProvTrust-WSN framework.

Dataset	Attack Type	Number of Events	Detection Rate
WSN-DS	Blackhole	79	1.000
WSN-DS	Flooding	81	1.000
WSN-DS	Grayhole	106	1.000
WSN-DS	TDMA	82	0.890
WSN-BFSF	Blackhole	113	0.920
WSN-BFSF	Flooding	285	1.000
WSN-BFSF	Forwarding	77	0.935

**Table 11:** Ablation study of the proposed PhyProvTrust-WSN framework.

Configuration	Accuracy	Precision	Recall	F1-score	FPR
Full PhyProvTrust-WSN	0.994	0.974	0.974	0.974	0.003
Without provenance reasoning	0.989	0.963	0.964	0.963	0.008
Without trust propagation	0.986	0.956	0.958	0.957	0.011
Without physics validation	0.981	0.944	0.946	0.945	0.017
Anomaly detection only	0.972	0.931	0.934	0.932	0.026

**Table 12:** Summary statistics of trust evolution and risk fusion during the experimental evaluation.

Metric	Observation
Initial average trust	High trust assigned to all sensor nodes
Trust trend under attacks	Gradual decrease proportional to accumulated evidence
Trust recovery	Supported after sustained benign behavior
Risk evolution	Smooth increase with accumulated evidence
Decision stability	High; limited oscillation around thresholds
False alarm suppression	Improved through multi-factor evidence fusion

**Table 13:** Communication overhead comparison.

Method	Relative Communication Overhead	Improvement Compared with Full Blockchain
ML-only IDS	1.00	37.5%
Trust-only	1.05	34.4%
Provenance-only	1.30	18.8%
Full Blockchain	1.60	—
PhyProvTrust-WSN	1.18	26.3%

**Table 14:** Scalability evaluation of PhyProvTrust-WSN.

Number of Sensor Nodes	Average Latency (ms)	Average Energy (mJ/Event)
100	9.4	0.22
200	12.7	0.27
400	17.5	0.36
600	22.3	0.43
800	27.9	0.50
1000	32.5	0.59

## Data Availability

The datasets used in this study are publicly available through their original sources. The retained experimental archive contains the reported performance results; however, the original run-configuration files containing calibrated parameter values were not preserved and are therefore not currently available. If recovered, these files will be released as original configuration files to support full reproducibility.
